# Modeling of the immune response in the pathogenesis of solid tumors and its prognostic significance

**DOI:** 10.1007/s13402-020-00519-3

**Published:** 2020-06-02

**Authors:** Łukasz Zadka, Damian J. Grybowski, Piotr Dzięgiel

**Affiliations:** 1 Division of Histology and Embryology, Department of Human Morphology and Embryology, Wroclaw Medical University, ul. Chalubinskiego 6a, 50-368 Wroclaw, Poland; 2 Orthopedic Surgery, University of Illinois, 900 S. Ashland Avenue (MC944) Room 3356, Molecular Biology Research Building Chicago, Chicago, IL 60607 USA

**Keywords:** Cancer, immune system, immunosuppression, inflammation, solid tumor, immune response

## Abstract

**Background:**

Tumor initiation and subsequent progression are usually long-term processes, spread over time and conditioned by diverse aspects. Many cancers develop on the basis of chronic inflammation; however, despite dozens of years of research, little is known about the factors triggering neoplastic transformation under these conditions. Molecular characterization of both pathogenetic states, i.e., similarities and differences between chronic inflammation and cancer, is also poorly defined. The secretory activity of tumor cells may change the immunophenotype of immune cells and modify the extracellular microenvironment, which allows the bypass of host defense mechanisms and seems to have diagnostic and prognostic value. The phenomenon of immunosuppression is also present during chronic inflammation, and the development of cancer, due to its duration, predisposes patients to the promotion of chronic inflammation. The aim of our work was to discuss the above issues based on the latest scientific insights. A theoretical mechanism of cancer immunosuppression is also proposed.

**Conclusions:**

Development of solid tumors may occur both during acute and chronic phases of inflammation. Differences in the regulation of immune responses between precancerous states and the cancers resulting from them emphasize the importance of immunosuppressive factors in oncogenesis. Cancer cells may, through their secretory activity and extracellular transport mechanisms, enhance deterioration of the immune system which, in turn, may have prognostic implications.

## Introduction

The immune system in mammals is strictly structured on the tissue level, which includes central and peripheral immune organs, as well as on the cellular level, which is based on the highly specialized properties of different cells. Such an organized system allows the establishment of an efficient defense against foreign environmental factors or endogenous antigens interpreted as extraneous [1]. Effective communication at the cellular and molecular level ensures the balance between activation of the immune response and its silencing [2].

During the development of cancer, immunological homeostasis is disturbed. On the one hand, some tumors increase immunopathological activity, predisposing them to the occurrence of paraneoplastic syndromes by the release of highly immunogenic factors, which leads to dysfunction of specific organs [3]. On the other hand, tumor promotion is usually accompanied by an increased frequency of infection due to the occurrence of secondary immune deficits, which is also associated with a more severe course of infection and an increased mortality among treated patients [4–7]. A separate issue that should be emphasized is the importance of chronic inflammation in the pathogenesis and progression of numerous cancers [8–10]. Additionally, some extracorporeal factors (such as a diet activating the immune system) may have a pro-carcinogenic effect that should not be underestimated [11, 12].

The changed parameters of the immune response seem to have increasingly wider clinical application, allowing us to show a correlation with survival curves and prognostic potential for oncological patients. The preoperative systemic immune-inflammation index (SII) score, describing systemic inflammatory activity, shows predictive value in tumors after surgical resection. It corresponds with a worse prognosis and more local aggression and has been associated with a more advanced clinical stage of cancer [13]. In advanced neoplasms, the number of circulating cancer cells is dependent on the stage of the tumor and inflammatory indexes [14]. The immunoscore system based on quantitative assessment of cytotoxic and memory T cells present in both neoplastic tissue and inflammatory infiltration of the marginal zone of cancer emphasizes the importance of immunological status in the prediction of the prognostic value of some solid tumors [15].

In 1957, Sir Macfarlane Burnet and Lewis Thomas proposed the basis of an immune surveillance theory, according to which transformation of cancer cells can occur in the human body and be effectively controlled by assuming an effective immune response against newly formed antigens on the surface of these cells, in which a significant contribution of lymphocytes was speculated [16]. Carcinogenesis affects the function of the immune system, with particular emphasis on the effector functions of T cells. Necrotic tumor cells release antigens that are processed by antigen-presenting cells (APCs). Antigen presentation to T cells activates them and allows tumor recognition and response [17]. The currently proposed concept of “Cancer Immunoediting” assumes a mutual influence of the host's immune response on the tumor burden and the ability to regulate this response during oncogenesis. Cancer cells modulate the functions of immune cells surrounding the tumor and the tumor microenvironment (TME), avoiding the effects of the antitumor immune response and determining the subsequent progression of the disease [16]. One example of pro-tumoral activity is the release of numerous suppressors of the host immune response, which changes the immunophenotype of immune cells, abolishes effector functions of lymphocytes and impairs the cytotoxic capabilities of CD8^+^ T cells [17].

Despite the obvious importance of immune surveillance against neoplastic transformation, the role of the immunologic system in cancer is not always clear. In primary immunodeficiencies, cancer risk is higher than in the general population, however the spectrum of emerging cancers is narrow and usually refers to changes originating from T cells. The limited role of the immune system in neoplastic transformation may be explained by the generally low antigenicity of tumor cells. Surface antigens on tumor cells rarely induce a strong immune response, and in the neonatal period (when the immune system is poorly developed) some tumors have a good prognosis [18]. The aim of this review is to evaluate the immunosuppressive effects induced by cancer on the parameters of the host immune response and to discuss their potential significance in the pathogenesis and clinical aspects of solid tumors, with a particular focus on chronic inflammatory-associated cancers.

## The most common inflammation-associated risk factors for cancer and their impact on tumor initiation

Despite many studies carried out so far, little is known about the pathogenetic conditions triggered in neoplastic transformation based on inflammation. Due to the complexity of the phenomena, there is also insufficient evidence to fully determine the significance of the immune response and its individual parameters/biomarkers. In terms of epidemiological data, some of the risk factors common to the cancers discussed in this review may lead to changes in immunological parameters and induce local inflammation. Prolonged exposure to a factor triggering the activation of the immune system can lead to subsequent cell damage and persistent functional-structural changes both at local and systemic levels, which in turn may predispose patients to the development of cancer [19].

The incidence rate of some cancers increases with age. The aging process seems to induce mild inflammation, which in the long-term disrupts the functionality and proportions of the cellular responses. In the elderly, some proinflammatory cytokines are overexpressed, such as interleukin-6 (IL-6), and T cells change their number. Simultaneous modifications of lymphocyte cell surface antigens, disproportion between T helper 17 (Th17) and regulatory T (Treg) cells and promotion of the Th2 response lead to functional changes in the immune system [20]. During aging there is also a redistribution of adipose tissue and changes in the composition and abundance of bacterial flora of the gastrointestinal tract. Hypertrophy of adipose tissue is associated with infiltration of immune cells secreting cytokines modifying glucose and lipid metabolism, which leads to the acquisition of insulin resistance [21]. Hence, type II diabetes is a known risk factor for pancreatic cancer (PC) and hepatocellular carcinoma (HCC) [22, 23].

Eating habits also remain a related risk factor. Some food ingredients can modify the immune response and change the level of proinflammatory factors. A diet rich in animal fat and meat increases the risk of colorectal cancer (CRC). It is believed that both components of food are associated with the induction of metabolites with carcinogenic activity [24]. A high content of fats in the diet shows a relationship with increased gut permeability and inflammation of adipose tissue [21]. Both alcohol consumption and smoking increase the risk of CRC, HCC and PC [23–26]. Long-term smoking has a toxic effect on the immune system, predisposing it to chronic low-grade systemic inflammation [27], while chronic alcohol consumption is associated with a disruption of cellular responses by lowering natural killer (NK) cells and T lymphocytes along with a change in cytokine expression compared to normal controls [26].

Factors triggering the local inflammatory milieu lead in initial stages to increased blood flow, vasodilation and migration of leukocytes from the vascular bed, which constitute an essential component of infiltration. Under normal conditions, processes triggering immune system activation disappear to enable the immune resolution process and the restoration of tissue functionality [28]. At this stage, there is some balance between leukocytes migrating to the target site and infiltrating the tissue and inflammatory cells undergoing apoptosis and leaving the infiltrate.

**Fig. 1.**
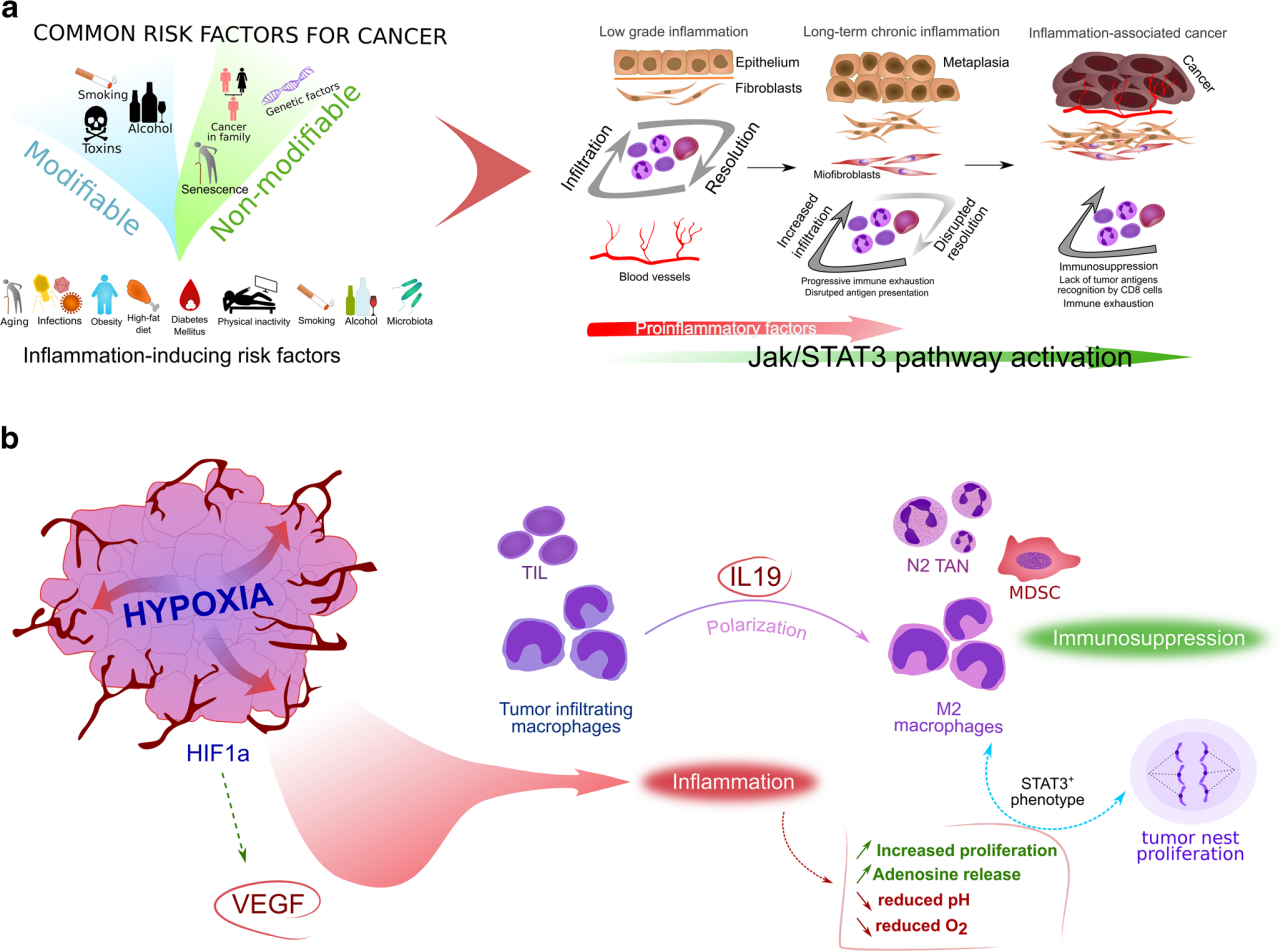
**A proposed simplified model of the association between inflammation and oncogenesis. A**. Common risk factors for cancers described in the text and their proinflammatory potential. Inflammation can be triggered by risk factors for cancer and long-term exposure to these factors can lead to permanent changes in cell structure and signaling associated with neoplastic transformation. **B** Hypoxia and proangiogenic activity are responsible for modifying the tumor microenvironment. The secretory activity of cancer cells leads to a change in the polarization of immune cells, which increases immunosuppression and causes cancer progression

During long-term tissue exposure to antigen stimulation, there is a disturbed balance between immune cells, i.e., T cells at the target site tend to remain and accumulate [29]. Macrophages migrating to the site of infiltration phagocytose certain cells and during chronic conditions secrete increased amounts of immunosuppressive factors, such as interleukin-10 (IL-10) and transforming growth factor-β (TGF-β). The second cytokine also acts as a growth factor, under the influence of which some mesenchymal cells may acquire features of smooth muscle cells, becoming myofibroblasts whose task is, among others, overproduction of extracellular matrix components [30, 31]. The local extracellular microenvironment and stroma components in chronic inflammation support the retention and increased survival of immune cells [29]. As shown in Figure 1, during chronic inflammation a disrupted cellular response occurs, accompanied by a pronounced tendency towards immunosuppression by overexpression of IL-10 and phosphorylation of signal transducer and activator of transcription 3 (STAT3). The potential proliferative effect of STAT3 on fibroblasts presented in our model may further support the chronicity of inflammation through improper accumulation of leukocytes in the infiltrate dependent on the secretory activity of mesenchymal cells. STAT3 regulates the inflammatory processes associated with the induction of metaplasia outbreaks, increases epithelial cell proliferation and appears to be responsible for the development and progression of some cancers [32]. In our previously published studies, the interleukin 10 receptor alpha subunit (IL10RA), IL-10 and phosphorylated STAT3 showed a statistically significant positive correlation with the cell proliferation antigen Ki67 in CRC [33], which seems to confirm the importance of Janus kinase and signal transducer and activation of transcription (JAK-STAT) signaling in the pathogenesis of some tumors (Fig. 1A).

## Cellular elements of tumor immunity

### Tumor infiltrating lymphocytes and their prognostic value in clinical outcome

A particularly important role in the anti-cancer immune response is played by lymphocytes whose effector functions allow reduction in tumor invasion. On the other hand, during carcinogenesis, the secretory activity of tumor cells can lead to suppression of the host's cell-mediated immunity. The molecular basis of these relationships has a significant impact on the clinical course of the disease because disturbed T cell function contributes to the progression of some cancers [34]. The exact characterization of tumor-infiltrating lymphocytes (TILs) can even have therapeutic relevance. Neoantigen-specific immune reactivation of TILs, unique for each patient, is currently considered a method of personalized anticancer treatment [35].

In breast cancer, TILs represent an immunological parameter whose morphological characteristics are of clinical significance. This parameter is related to the histopathological-molecular type of the tumor and may be an independent prognostic biomarker of e.g. the response to adjuvant chemotherapy [36]. Cancer-induced changes in gene expression and T cell receptor (TCR) activity in the CD8^+^ cell population modify TIL density, which determines the response to anticancer treatment and the prognosis of patients, and may be a promising starting point for therapy [37]. Evaluation of inflammatory immune infiltration in cancer may also reflect the defense capabilities of the immune system. The density of immune cells in cancer illustrates the severity of the host antitumor immune response and usually refers to clinical aspects of the disease.

In CRC, the assessment of CD8^+^ T cell density in randomly chosen invasive tumor margin infiltrations has shown a significant relationship with survival [38]. In CRC, TILs are a favorable prognostic parameter – a high number of immune cells was associated with statistically better overall survival (OS**)** values than was a low tendency toward lymphocytic infiltrates [39]. The distribution of immune cells in tumor tissue may also have prognostic significance. In esophageal squamous cell carcinoma, TILs showed redistribution of the location in favor of the stroma rather than the intraepithelial location, and the higher intensity of infiltration in the stroma showed prognostic advantage [40]. Nevertheless, the clinical significance of inflammatory immune infiltration can have a different outcome depending on the type of cancer. More pronounced lymphocytic infiltrates are characteristic for lobular breast cancer (LBC) rather than for invasive ductal cancer (IDC), and high levels of TILs have been associated with tumor cell proliferation, with more frequent lymph node infiltration and a worse prognosis [41]. In breast cancer, the prognostic value of TILs also appears to depend on the molecular specifications of tumor cells. An increased TIL concentration is a beneficial prognostic marker for human epidermal growth factor receptor 2 (HER2)-positive breast cancer and TNBC (triple-negative breast cancer), but is unfavorable for luminal HER2-negative breast cancer [42]. A significant prognostic role is played by the immunophenotype of tumor-infiltrating immune cells. The prognostic value of TILs depends on the specification of the lymphocyte immunophenotype – the predominance of T cells promoting cellular cytotoxicity and APCs is usually a favorable parameter [43]. In the case of extrahepatic cholangiocarcinoma, a beneficial prognosis is associated with the presence of memory cells with a CD8^+^CD45RO^+^ phenotype in the lymphocytic infiltrate, but not with CD8^+^ only cells [44]. The predominance of CD4^+^ T cells in tumor-infiltrating immune cells of CRC metastases to the lungs has been found to be associated with a better outcome after surgical resection [45].

### Tumor associated macrophages

Tumor associated macrophages (TAMs) residing in neoplastic tissue constitute a fairly heterogeneous population of cells. Some cells are derived from monocytes circulating in the blood, the reservoir of which is the bone marrow. The process of migration occurs through chemotaxis and is conditioned by tumor-released ligands, such as chemokine (C-C motif) ligand 2 (CCL2), CCL5 and colony stimulating factor 1 (CSF-1). A distinct subgroup is a population of TAMs derived from embryonic precursors that colonize the tissue at early stages of ontogenesis [46]. Various subpopulations of macrophages with different regulatory effects on the immune response can be distinguished in the population of monocytes. The subpopulation of M1 macrophages has proinflammatory properties, evokes a Th1-type response, and has strong cytotoxic activity as part of the antitumor response. Subpopulations of M2 macrophages usually have immunoregulatory properties, are responsible for a Th2-type response and produce small amounts of pro-inflammatory cytokines. M2 TAMs secrete immunosuppressive factors, such as IL-10 and TGF-β. In the M2 subpopulation, as many as 3 major subtypes of macrophages with different functions and secretory profiles can be distinguished: M2a, M2b and M2c macrophages [47]. There are also M2d macrophages, characterized by high levels of vascular endothelial growth factor (VEGF), IL-10 and cytokine-inducible nitric oxide synthase (iNOS) expression [48].

The subpopulation of M2 macrophages is a common element of the TME and is usually an unfavorable prognostic factor in various types of cancer. Their activity has been associated with greater invasiveness, shorter disease-free survival and induction of epithelial-mesenchymal transition (EMT) [49]. Neoplastic cells induce polarization of M0 macrophages towards the M2 phenotype. On the other hand, physical properties of the cellular matrix may increase tumor invasiveness by regulating the transcription of EMT-related genes in the population of M2c macrophages [50]. TNF-α-releasing M2 macrophages promote the progression of HCC and induce EMT, which is associated with regulation of the Wnt/β-catenin pathway by cytokines [51]. In HCC, upregulation of Wnt/β-catenin signaling depends on Notch signaling. In Kupffer cell-like TAMs (kclTAMs), the process increases cell proliferation, downregulates interleukin-12 (IL-12) synthesis and increases IL-10 release. This in turn leads to the progression of HCC and promotes the potential of some solid cancers to form metastases in the liver - however, this potential is dependent on the type of cancer [52]. Other immunosuppressive cytokines released by TAMs may also lead to the progression of solid tumors. A higher number of TAMs in CRC is associated with a tendency to form distant metastases. Greater invasiveness and migration of cancer cells results from the regulation of EMT through TGF-β secreted by TAMs via the Smad2,3-4/Snail/E-cadherin pathway [53]. Overexpression of NOR1 observed in HCC has been associated with an unfavorable prognosis, which is most likely due to acceleration of the immunosuppressive activity of NOR1^+^ TAMs by upregulation of arginase 1 (Arg1) expression and preferred polarization of macrophages towards the M2 subpopulation [54]. Human prostate cancer xenografts showed a clear polarization of the macrophage population towards the M2 phenotype - the higher TAM density stimulated the growth of neoplastic lesions. Moreover, CD206 macrophages were more likely to settle in strongly vascularized regions characterized by co-expression of the CD31 antigen, which may suggest the importance of TAMs in the regulation of angiogenesis [55]. Hyperactivity of M2 macrophages is also typical of some subtypes of inflammatory cancers. An inflammatory infiltrate formed by M2 macrophages is relatively common in inflammatory breast cancer (IBC). In this type of cancer, TAMs clearly overexpress interleukin-8 (IL-8) and growth-regulated oncogene (GRO) chemokines, which are associated with epithelial-to-mesenchymal transition CSC-like phenotypes through the activation of JAK2/STAT3 signaling [56]. Overexpression of some inflammatory factors may paradoxically increase promotion of the immunosuppressive phenotype. In breast cancer (basal-like BCC), the S100 calcium-binding protein A4 (S100A4) promotes monocyte differentiation and polarization towards M2 phenotype macrophages as well as induces increased expression of interleukin-6 (IL6) and IL8 in macrophages [57].

TAMs have an adverse effect on cellular responses, especially on the action of T cells. Tumor cells capable of releasing CXCL2 ligand activate CXCR2^+^ TAMs, enhancing their suppressive effect on T cells and pro-angiogenic activity, with consequent tumor growth [58]. TAMs also impede the migration of CD8^+^ T cells into the tumor nests as well as restricted inflammatory infiltration by these cells. This significantly determines the effectiveness of cancer immunotherapy [59]. An interesting observation is also the relationship between the molecular profile of TAMs and TILs. In pancreatic ductal adenocarcinoma (PDA), a significant relationship has been observed between TAMs and TILs, where different epigenetic profiles of macrophages influenced the regulation of the molecular parameters of T cells infiltrating the tumor. A reduced number of CD11b^+^ and CD115^+^ cells resulted in an increased number of T cells infiltrating the tumor, a lower number of IL-10-releasing CD4^+^ T cells and a smaller Treg population [60]. The regulation of the migration of TAMs into the tumor nest is also dependent on other factors. In non-small-cell lung carcinoma (NSCLC), the recruitment of macrophages relates to the expression of the VEGF-C cytokine, whose overexpression potentiates tissue infiltration by this population of cells. The ligand secreted by tumor cells activates the VEGFR-2 and VEGFR-3 receptors located on macrophages, leading to their increased migration through Src/p38 signaling [61].

Although most reports revealed adverse effects of TAMs on the process of oncogenesis, particularly related to the M2 subpopulation, there are also data suggesting a beneficial effect of these cells on prognosis. CD68^+^ cells are most numerous in the inflammatory infiltrate of CRC. Their prognostic value seems to depend on the microanatomical location of the infiltrate. Long-term follow-up showed a tendency of CD68^+^ macrophages to infiltrate the anterior regions of the invasive tumor as a favorable prognostic parameter [62]. In CRC, a tendency to induce expression of proinflammatory cytokines has also been reported. Exposure of peripheral blood mononuclear cells to conditioned medium from CaCo-2 cells changed the secretory activity of monocytes by promoting the release of proinflammatory cytokines, such as IL-6, IL-12 and interferon gamma (IFN-γ), with downregulation of the expression of some immunosuppressive interleukins, such as interleukin-4 (IL-4) and IL-10. The group of proinflammatory cytokines also revealed a reduced expression of TNF-α, which may result from an increased pro-angiogenic activity of tumor cells caused by negative regulation by VEGF [63].

M1 macrophages, whose secretory profile differs from the properties of the M2 population, play a different role in the pathogenesis of tumors. A tendency for an increased expression of TNF-α is typical of M1 macrophages, for which this cytokine remains a marker protein additionally to blocking polarization towards the M2 subtype [64, 65]. The favorability of macrophage repolarization to M1 cells has been associated with elevated levels of reactive oxygen species (ROS), a higher concentration of proinflammatory agents and a simultaneous decrease in the expression of CD206 and Arg1 [64]. Additionally, in this case, the M1 subtype is not always a favorable prognostic factor. In breast cancer, M1 macrophages are usually an unfavorable predictor. In vitro studies have shown that the pro-inflammatory secretory activity of M1 macrophages may increase invasiveness by promoting the EMT phenotype. The regulation of this phenomenon is complex and strictly dependent on other molecular parameters of the cancer cells. Bednarczyk et al. noted a positive effect of increased matrix metallopeptidase 9 (MMP-9) expression on the migrative and invasive potential of adenocarcinoma cells induced by the M1 subpopulation [66].

### Tumor associated neutrophils

The heterogeneity of the tumor-associated neutrophil (TAN) population has not been sufficiently defined and, therefore, requires more detailed studies. Nevertheless, morphologically their population is divided according to differences in density gradient purification, where low-density neutrophils (LDNs) can be distinguished, which are most often associated with cancer progression, as well as a population of so-called high-density neutrophils (HDNs), which comprises highly diverse and mature inflammatory cells. Alternatively, N1 and N2 neutrophils may be distinguished, characterized by different gene expression profiles and secretion parameters. TAN polarization characterized by the N2 phenotype, which is identified as promoting oncogenesis, takes place under the influence of TGF-β. In contrast, the N1 phenotype is characterized by lower Arg1 levels, distinctive anticancer activity and a tendency toward cytokine and chemokine expression, which stimulate an inflammatory response [67, 68]. Within the cell response range, the N2 phenotype regulates CD4^+^ T cell recruitment, whereas N1 TANs seem to be responsible for cytotoxic functions. In the division that concerns cancer-related circulating neutrophils, the LDN phenotype limits the ability to perform phagocytosis and exhibits antiproliferative activity toward CD8^+^ T cells, whereas HDN cells have antagonistic properties [69].

The neutrophil-to-lymphocyte ratio (NLR) is a key prognostic factor in various cancer types and is relatively frequently correlated with recurrence risk [70–72]. A high NLR in bladder cancer positively correlates with determinable concentrations of IL-6 and IL-8 as well as with their Treg expression in peripheral blood, which emphasizes a systemic dependence between neutrophil infiltration in cancer and the secretion profile of cytokines. Furthermore, higher concentrations of both cytokines, of prevailing proinflammatory activity, have shown a positive relation to Treg induction. In contrast, high NLR values have shown a positive correlation with tumor stage, tumor growth and the level of common inflammatory markers such as C-reactive protein (CRP), the plate-to-lymphocyte ratio (PLR), and the monocyte-to-lymphocyte ratio (MLR) [73].

The presence of TANs in inflammatory infiltrations most often disturbs the accumulation and activity of T cells, which is related to clinical and pathological data. In patients with extrahepatic cholangiocarcinoma, TANs showed an inverse correlation to CD8^+^ T cells and a strong association with a poor overall survival (OS) [74]. Myeloperoxidase (MPO)^+^ neutrophils releasing interleukin-17 (IL-17) are a favorable prognostic factor in esophageal squamous cell carcinoma (ESCC). It was confirmed in in vitro models that a high percentage of immune cells releasing IL-17 correlated with the release of CXCL2 and CXCL3 ligands, which resulted in cells having a greater tendency toward migration [75]. On the other hand, it has been shown that T cells releasing IL-17 may be linked to cancer progression, and that TANs may show properties that enhance anticancer responses via γδ T cell suppression due to oxidative stress intensification and ROS production [76]. However, under restricted glucose conditions, ROS production in the mitochondria of neutrophils may lead to T cell suppression, which links oxidative stress to increased immunosuppression [77].

### Myeloid-derived suppressor cells

Myeloid-derived suppressor cells (MDSCs) represent a group of heterogeneous subpopulations of immature myeloid cells with predominant immunosuppressive properties that are widespread in various types of human cancers, as well as in mouse tumor models. In tumor-bearing mice, the expression of relevant molecules enables the classification of MDSCs into two main subtypes: monocytic lineage cells (mo-MDSCs) and granulocytic (polymorphonuclear) lineage cells (PMN-MDSCs). Three subgroups of these cells are distinguished in humans depending on their immunophenotype and immunosuppressive properties: PMN-MDSCs, M-MDSCs and Lin^-^HLA^-^DR^-^CD33^+^ cells [78]. Cancer cells and immune cells have the potential to induce the formation of MDSC subtypes. Human T cells are of pivotal importance in inducing a population of PMN-MDSCs. Through a direct cell-cell mechanism with the use of the transmembrane form of TNF-α (tmTNF-α) activation, CD4^+^ T cells promote the development of a PMN-MDSC population using CD33^+^ myeloid cell reserves, while the CD3^+^ subpopulation shows a correlation with anti-apoptotic activity toward PM-MDSCs [79]. Cancer cells release CXCL1 and CXCL2 chemokines, which in turn induce the generation of mo-MDSCs as a bone marrow cell subpopulation [80].

In patients diagnosed with renal clear cell carcinoma (RCC) an estimatable level of total MDSCs, granulocytic MDSCs (G-MDSCs), and immature MDSCs (I-MDSCs) clearly correlates with the degree of histological malignancy and stage of the disease, whereas stromal MDSCs positively correlate with IL-17 and IL-18 co-expression, both at the protein and mRNA level. An increased level of the two interleukins in relation to MDSCs was also noted in peripheral blood, i.e., PBMCs. The MDSCs and cytokine concentrations were significantly higher in RCCs than in controls [81]. The preoperative number of PMN-MDSCs and mo-MDSCs tend to be different depending on TNM breast cancer stage. The lowest percentages of mo-MDSCs were noted in stage Tis (in situ), whereas a higher number of these cells was observed in the 3rd stage of the disease. In the case of PMN-MDSCs, however, the dependency was reversed, with stage Tis being characterized by the highest percentage of this MDSC subpopulation [82]. The number of MDSCs in an ovarian cancer mouse model increased with neoplastic process duration and was significantly higher in late stages of the disease. The significant immunosuppressive activity of MDSCs directed against T cells leads to disturbed systemic immunity in animals [83].

The epithelial-to-mesenchymal transition phenomenon in ovarian cancer is related to the Snail transcription factor, whose expression level is significantly correlated with a lower percentage of survival. Snail also shows a correlation with a higher percentage of intratumor MDSCs, whose number reduces the CD8^+^ population in TILs. As demonstrated in a mouse model, MDSC migration toward tumor tissue takes place by chemotaxis and depends on chemokines such as CXCL1 and CXCL2. High levels of these chemokines were noted in cancers with a positive Snail co-expression. The use of a CXCR2 ligand antagonist limited MDSC migration, and the chemokine receptor alone was associated with the promotion of tumor growth and an unfavorable prognosis. Nuclear factor kappa B (NF-κB) has been reported to participate in Snail-dependent regulation of CXCL1 and CXCL2 expression [84]. Chemotaxis dependent on relevant chemokine expression appears to be important, not only in MDSC migration toward the primary tumor, but also in the promotion of distant metastases and upward regulation of growth factors. Breast cancer cells that secrete CXCL17 increase lung CD11b^+^Gr^-^1^+^ MDSC accumulation and increase the level of platelet-derived growth factor-BB (PDGF-BB) expression in these cells, which is also associated with proangiogenic activity that facilitates lung metastatic niche formation [85]. The chemokine expression level supporting intratumor MDSC recruitment has been found to be regulated by transcription factors. A high level of ΔNp63 in triple-negative breast cancer (TNBC) is positively correlated with the size of the MDSC population, which represents a direct ΔNp63-dependent activation of chemokines such as CXCL2 and CCL22 [86]. CXCR2^+^ PMN-MDSCs enhance tumor growth, whereas their migration from the periphery to the tumor appears to be dependent on CXCR2. PMN-MDSCs are characterized by pronounced TIL suppression, which may be accompanied by programmed death (PD)-axis signaling. Due to the capability of regulating tumor T cell infiltration, the PMN-MDSC subpopulation may have considerable significance in cancer immunotherapy [87].

In the regulation of carcinogenesis, MDSCs are crucial not only at advanced stages, but also in early precursor lesions of malignant cancers. In premalignant lesions, the amounts of MDSCs have been found to be considerably greater than those in controls. Its amounts were, however, lower than those in overt cancers, whereas the immunosuppressive activity of these cells was comparable in precancerous and cancer stages [88]. The transcription profile of peripheral blood MDSCs (PB-MDSCs) reflects molecular changes appearing in different stages of tumor development. Upregulated genes such as those encoding Arg1 and nitric oxide synthase 2 (NOS2) seem to confirm an immunosuppressive tendency of tumor progression. Mammalian target of rapamycin kinase (mTOR) pathway activation and Toll-like receptor (TLR), IL-4, IL-6 and IL-10 signaling take place in early stages. In patients with gastric cancer (GC), IL-6 and IL-8 activate CD45^+^CD33^low^CD11b^dim^ MDSCs, thereby inducing Arg1 release accompanied by activation of the PI3K-AKT signaling pathway. Activation of this pathway suppresses CD8^+^ T cells and positively correlates with disease progression and patient survival rates in general. Increased levels of IL-6 and IL-8 in patients with GC have been found to positively correlate with Arg1 and MDSCs [89]. There are reports stating that Arg1 is not consecutively released by MDSCs and that its synthesis depends on the activity of certain interleukins, i.e., IL-6, IL-4, GM-CSF and IL-10 are known to regulate the secretion of Arg1 by MDSCs.

The induction of Arg1 synthesis seems to be indirectly regulated, with IL-6 regulating IL-4R receptor expression on MDSCs, which subsequently enhances Arg1 synthesis by binding to the ligand. In the second option, GM-CSF induces IL-10R expression, and the binding of the cytokine to the receptor results in Arg1 release [90]. Polymorphonuclear myeloid-derived suppressor cells (PMN-MDSCs) in prostate cancer together with Arg1, NOS2 and STAT3 expression, exhibit a suppressive effect on T cell activity. PMN-MDSC suppressive activity seems to be regulated through interactions of these cells with CD40L^+^ mast cells resulting from CD40 ligand co-stimulation. This correlates with the induction of oncogenesis in transgenic adenocarcinoma of the mouse prostate (TRAMP) and affects cell immunity [91]. In epithelial ovarian cancer (EOC) a positive correlation has been observed between TGF-β, estimable in plasma, and Arg1^+^ MDSCs found in the peritoneal fluid. The plasma level of Arg1 was also found to be positively correlated with TGF-β^+^/IDO^+^/IL-10^+^ PMN-MDSCs [92]. The immunosuppressive tumor microenvironment is an important regulator of the response to radiotherapy. Indoleamine 2,3-dioxygenase 1 (IDO1) inhibition within the range of IDO1-expressing myeloid-derived suppressor cells in a mouse model of lung cancer decreased the size of the population of these cells, increased the immunosuppressive effect and sensitized the tumor to radiation [93]. Expression of programmed death 1 (PD-1) on the MDSC surface is regulated by the NK-κB signaling pathway and is increased by MDSC tumor infiltration. The presence of PD-1^+^ MDSCs in the TME is associated with acceleration of their proliferation in relation to PD-1^-^ MDSCs, which suggests that TME immunosuppressive properties may contribute to the promotion of carcinogenesis [94].

The use of STAT3 inhibitors in prostate cancer limited tumor-associated MDSCs and inhibited interleukin-1β (IL-1β), IL-10 and IL-6 synthesis by monocyte cultures [95]. STAT3 inhibition in liver-associated MDSCs (L-MDSCs) has been found to exhibit antitumor activity, whereas a decreased level of STAT3 phosphorylation also reduced the size of the L-MDSC population, which in turn led to an increased anticancer activity of chimeric antigen receptor T cells (CAR-T) [96]. MDSCs generated from induced pluripotent stem cells (iPSCs) in an autoimmune hepatitis murine model disturbed the cellular response while limiting lymphocyte proliferation and CD8^+^ T cell inflammatory infiltration in portal tract regions, which was accompanied by significantly decreased alanine aminotransferase levels (ALT) in plasma [97]. In the case of CRC, the number of MDSCs showed a discernible disturbing effect on the cellular response associated with lymphocyte redistribution toward the monocyte line resulting in a higher level of circulating MDSCs, which was correlated with a lower lymphocyte to monocyte ratio (LMR). A significantly decreased LMR was also found to be associated with a reduced recurrence-free survival [98]. Abundant MDSC infiltration in osteosarcoma also resulted in increased T cell cytotoxic activity, while its accumulation in cancer tissue appeared to be dependent on expression of the CXCR4 receptor, which enabled the migration of these cells by stromal cell-derived factor 1 (SDF-1). Moreover, binding of this chemokine with CXCR4 inhibited MDSC apoptosis and was inversely correlated with infiltration of the cancer by CD8^+^ T cells. CXCR4 blockade, on the other hand, in combination with anti-PD-1 therapy exhibited a synergistic effect [99]. In CRC, MDSC function is regulated via RIPK3-PGE2. MDSC accumulation has been found to decrease the level of RIPK3, which enhanced oncogenic potential via the promotion of MDSC accumulation and increased immunosuppressive activity resulting from NF-κB upregulation, which regulated COX2 transcription and inhibited prostaglandin E2 (PGE2) release, thereby stimulating cancer cell proliferation while negatively influencing CD8^+^ T cells [100].

The number of MDSCs in patients with breast cancer has been found to be higher than that in the control group and to correlate with both the tumor size and the stage of the disease. The IL-17 level, on the other hand, was found to be lower in the patients than in the controls [101]. Proinflammatory IL-17 showed an anti-proliferative effect on MDSCs and induced their differentiation, which was accompanied by an altered expression profile of specific cytokines. Under the influence of IL-17, MDSC release lowered the TGF-β and IL-10 levels, whereas the IL-1, IL-1β, IL-6 and TNF-α levels were upregulated. Ma et al. observed a correlation between MDSC accumulation inhibition by IL-17 and STAT3 activation [101]. In lung cancer, the TLR1/TLR2 expression levels served as a favorable prognostic factor. In an animal model, TLR1/TLR2 activation was found to be linked to a reduction in tumor growth and a selective downregulation of the M-MDSC subpopulation. The use of a TLR2 agonist exhibited a positive effect on M-MDSC redistribution toward M1 macrophages [102].

## The role of cytokines in immune regulation during carcinogenesis

Cytokines constitute a large group of proteins with multipotent properties that play a role in regulating numerous cellular signaling pathways. The main sources of cytokines are leukocytes, but their secretion also occurs in other cells [103]. Germinal cells, for example, show the capability of secreting cytokines, including interleukins (ILs), which modulate antitumor immunity [104, 105]. Elevated levels of proinflammatory cytokines have been reported in many cancers and to be correlated with an increased risk of cancer, cancer progression, clinical stage and response to treatment [106, 107]. On the other hand, immunosuppression has also been associated with the induction of cancer [108]. Some cytokines with known suppressive effects on the host immune response, such as IL-10 and TGF-β, have been reported to have oncogenic potential [109–111]. The model for classification of cytokines released by immune cells considering the main effect of these molecules on the immune response is based on subgroups of CD4^+^ T helper cells (Th). Th1 cells release cytokines with predominantly proinflammatory effects, including IFNγ, IL-2, IL-12 and TNF-α. Th2 cells are immunosuppressive due to the release of IL-4, IL-5, IL-6, IL-10 and IL-13, while TGF-β as an immunosuppressant is mainly released by Tregs. Another subgroup, Th17 cells, plays an important regulatory role in autoimmunity and in allergic reactions and is characterized by the production of IL-21, IL-22 and IL-26 [112]. This breakdown does not seem to be optimal. Despite being assigned to Th2 cytokines, IL-6 usually exhibits proinflammatory rather than immunosuppressive activity. Moreover, both IL-6 and IL-10, despite significant functional differences, lead to activation of similar signaling pathways resulting from STAT3 phosphorylation.

### IL-6 cytokine family

A characteristic feature of the IL-6 family of cytokines (IL-6, LIF, CNTF, CT-1, oncostatin M and IL-27) is a shared use of the gp130 β-subunit. However, it must be noted that IL-6 linkage to its receptor (IL6Rα) triggers homodimerization of gp130, while the other members of the IL-6 family induce the formation of a heterodimeric gp130 receptor complex [113]. In contrast to the immunosuppressive actions of IL-10, IL-6 is usually proinflammatory, and its synthesis is related to initiation of the early acute phase of inflammation, an increase in the release of acute phase proteins by hepatocytes in the liver, and the promotion of differentiation of naive CD4^+^ T cells [114]. Consequently, elevated concentrations of IL-6 have been observed in various diseases including autoimmune diseases and cancers, and the cytokine itself modulates the immune response during chronic inflammation [114]. Although IL-6 belongs mainly to the proinflammatory cytokines, its properties depend on the type of receptor pathway and target tissue involved. During trans-signaling, IL-6 forms a complex with the soluble form of its receptor, after which activation of other cells often causes a proinflammatory effect. Classic interleukin signaling resulting from the binding of IL-6 to IL6R usually leads to immunosuppression by activating the JAK-STAT pathway [115].

### IL-10 cytokine family

The family of IL-10 cytokines that shares similar traits, structures and gene coding sites includes the following proteins: IL-10, IL-19, IL-20, IL-22, IL-24 and IL-26 [116]. In terms of its best-known functions, IL-10 belongs to the major immunosuppressants that regulate immune responses, properties of B and T cells, and suppression of monocyte activity. It also acts on macrophages and inhibits the release of pro-inflammatory cytokines. Through interaction with the IL-10 receptor complex (IL-10R), IL-10 affects the transcriptional activity of thousands of different genes and its production under physiological conditions affects almost all leukocytes. IL-10 is, for example, synthesized by T and B lymphocytes, dendritic cells, mast cells, NK cells and Tγδ cells [117]. With respect to the other cytokines of the IL-10 family, their targets are mainly tissues and organs of non-immune origin, such as epithelia [118].

IL-10 acts as an important mediator of Treg suppression and can also promote the transformation of peripherally induced Tregs (iTregs) into a Forkhead box P3 (Foxp3)-positive phenotype via IL-10R-mediated STAT3 signaling. Additionally, IL-10 activity against iTregs has been found to lead to inhibition of PI3K/Akt signaling and increases in Fox protein O1 (Foxo1) activity, which plays an important role in iTreg differentiation [119]. During carcinogenesis, numerous complex biological processes take place that result in the accumulation and synthesis of many metabolites affecting the plasticity of the microenvironment of the developing tumor. One of them is adenosine, which under favorable conditions, enhances the biological effect of IL-10 on activation of STAT3 in M2c macrophages [120].

### STAT3

Despite many differences in their biological effect on the functioning of cells as a result of activation of their receptors, both IL-6 and IL-10 ultimately lead to a common goal, that is the activation of STAT3, which in the case of IL-6 has a proinflammatory effect and suppressive effects on the immune system during activation of the IL10R signaling pathway. [121]. The STAT family in mammals consists of seven well-known members: STAT1, STAT2, STAT3, STAT4, STAT5a, STAT5b and STAT6. The STAT3 transcription factor can be activated by proteins such as IL-6, EGF, G-CSF, IL-2 and IL-10 [122]. The STAT family enables signal transduction of many cytokines and their ligands. Numerous studies have shown that phosphorylation of STAT proteins results in their dimerization and translocation to the cell nucleus with subsequent transcriptional activity of the dimer. Nevertheless, STAT1 and STAT3 mediators have been found to exist in the form of homodimers even before phosphorylation by tyrosine kinases, and STAT dimers also appear in the cytoplasm and not only in the nucleus [123]. Two STAT3 isoforms have been described, which are characterized by quantitative differences in DNA binding. Both isoforms (STAT3α and STAT3β) show similar DNA binding strengths and rates of association/dissociation with DNA. STAT3β dimers, however, have a stronger stability than the α-isoform, which in vivo can influence their dephosphorylation rate [124]. STAT3 dimerization is a key process for its transcriptional activity since only the dimerized form can bind to DNA. Un-phosphorylated STAT3 (U-STAT3) can be found both in the cytoplasm and in the nucleus and, similar to activated STAT3 (pSTAT3), it may form dimers and display activity as a transcription factor, a phenomenon that can be observed in normal somatic cells as well as in tumor cells. The regulation of U-STAT3 dimer activity appears to be dependent on the type and site of the covalent bonds, as well as on the conformation of the polypeptides [125].

STAT3 activation directly regulates the expression of oncogenes and promotes the suppression of anticancer immune responses, which facilitates the expansion of cancer. An example of STAT3 regulation in the synthesis of immunosuppressive factors is its influence on the release of IL-10, and its overexpression is responsible for, among others, tolerance of dendritic cells in tumor stroma, inhibition of proinflammatory cytokine secretion or inhibition of macrophage and T helper cell functions [126, 127]. In the neoplastic process, production of IL-10 may result in a different outcome depending on the type of cancer, and its source is usually the same cancer cell or Treg [126]. Polarization of the Treg phenotype in tumor tissue seems to be strictly dependent on activation of STAT3 [128]. In breast cancer, promotion of the IL-10 induced immunosuppressive Treg phenotype depends on the stage of cancer, and during its formation other mediators are involved in addition to activated STAT3. An example is FoxP3, whose dimerization allows the formation of a bond to histone acetyltransferase 1 (HAT1) and subsequent translocation of the resulting complex to the nucleus, where addition of the dimeric form of STAT3 results in epigenetic changes in the IL-10 promoter region, thereby upregulating the expression of the gene [129].

### Hypoxia-inducible factor 1 dependent cytokines

At almost every stage of cancer development, vascularization dominates the outer circumference of the cancerous tissue [130]. When the rate of tumor cell proliferation surpasses that of apoptosis, hypoxic foci begin to arise in the center of the growing tumor [131]. Hypoxia-related centers of necrosis are among the most important inducers of angiogenesis at relatively early stages of cancer development through the production of the hypoxia-inducible factor-1α (HIF-1α) [132]. Hypoxia-induced angiogenesis of the tumor, due to an increased release of HIF-1α, leads to a significant overexpression of VEGF, which usually increases the average microvascular density in the tissue [131, 133]. The hypoxic state is also potentiated during cancer-induced inflammation, resulting in increased cellular metabolism and quick oxygen depletion [134]. During the early stages of cancer development, neoplastic tissue (excluding typical angiogenic phenotypes) remains mostly avascular after which HIF-1α-dependent release of VEGF is a key process promoting neovascularization. This process is also active at later stages of tumor expansion as the formation of structurally and functionally inappropriate vascularization incessantly fosters the induction of hypoxia [135].

Hypoxic conditions in mammalian cells mainly lead to an increased synthesis of placenta-derived growth factor (PDGF), placental growth factor (PlGF), VEGF-A, VEGF-B, VEGF-C and VEGF-D. VEGF receptors (VEGFRs) are located not only on endothelial cells but also on extravascular tissues, and the biological properties of these receptors are characterized by their varying affinity for ligands, different degrees of tyrosine kinase activity, preferred localization and spectrum of isoforms resulting from various forms of alternative splicing [134]. In the hypoxic environment of a tumor, VEGF seems to be only one of many cytokines secreted after induction of HIF-1α. An acidic tumor microenvironment can promote the secretion of proinflammatory cytokines. In an in vitro carcinogenesis model, exposure of L-02 cells to the carcinogen arsenite promoted glycolysis and increased the expression of proinflammatory cytokines such as IL-6, TNF-α and IL-8, which was accompanied by a high co-expression of HIF-1α [136].

The promotion of HIF-1α-dependent inflammation seems to be related to overexpression of proinflammatory cytokines. Mladenova et al. found that in a mouse colorectal cancer model, HIF-1α potentiated chronic inflammation in the proximal colon of mice during long-term administration of a non-steroidal anti-inflammatory drug, sulindac [137]. The relationship between hypoxia and inflammation seems to have an impact not only on the preferential secretion of proinflammatory factors. STAT3 is a known mediator of signaling pathways of various cytokines, both proinflammatory and immunosuppressive. In malignant peripheral nerve sheath tumors (MPNSTs), inhibition of STAT3 led to decreased wound healing, cell migration, invasion and tumor formation, while STAT3 knockdown inhibited HIF1-α, HIF2-α and VEGF-A expression [138]. In some solid tumors, the presence of lymphocytic infiltrations appears to be an important prognostic and predictive factor of the response to therapy [139]. In serous ovarian cancer, inflammatory infiltration with CD8^+^CD4^+^FoxP3^+^ cells, a high degree of tumor vasculature, and overexpression of VEGF were favorable prognostic factors [140]. HIF-1α also seems to be important in regulating the secretory activity of inflammatory cells. CD4^+^ T cells, for example, have the ability to release IL-22 under hypoxia through a HIF-1α-dependent mechanism [141].

It is important to distinguish the inflammatory-induced angiogenic phenotype from the angiogenic phenotype caused by hypoxia and ischemia. During inflammation-induced angiogenesis CC-chemokines (CCLs) are released, thereby promoting directional proliferation of endothelial cells and macrophage recruitment toward the inflammatory site, which in turn induces the synthesis of proangiogenic stimulators [142]. Inhibition of VEGF and HIF-1α activity abolishes the proangiogenic activity, whereas in angiogenesis induced by hypoxia or ischemia there is no such effect [83]. In addition to HIF-1α involvement, ischemic and hypoxic conditions lead to the activation of different transcription and growth factors that may increase the production of proangiogenic cytokines independently of HIF-1α [143]. In hypoxia caused by ischemia, both proangiogenic factors and selected immunological factors are activated. In order to compensate for ischemia, IL-19 stimulates M2 polarization of macrophages, enhances the release of VEGF-A, and potentiates the proangiogenic effect by silencing the synthesis of IL-12, a cytokine inhibiting vasculogenic functions in the spleen [144]. IL-19 also acts indirectly on macrophages which, as a result of polarization towards the M2 line, release proangiogenic factors [145]. The above mechanisms and their common complexity should be included in the conceptual assumptions of tumor-induced angiogenesis (Fig. 1B).

The obvious association between tumor-development and inflammation, which can be modulated by various cytokines, raises a relevant question: how and to what extent do cytokines affect the immune response during carcinogenesis? The role of cytokines in the regulation of the immune system during cancer development is briefly presented in Table 1 together with potential mechanisms underlying their action and function in the regulation of immune responses.

**Table 1:** Cytokines and their influence on tumor immunity

Type of cancer	Cytokine	Recognized role in the immune response	Cancer-associated immune response
Ovarian cancer (SKOV-3)	IL-6	One of the most potent proinflammatory cytokines; activation of the Src kinase family; activation of STAT transcription factors [146, 147]	Inducing polarization of M2 macrophages [148]
Breast cancer (MCF-7)			Cellular senescence phenotype [149]
Tumor-derived murine squamous cell carcinoma cell line (PDSC5)/ fibroblasts accelerate stromal supported tumorigenesis (FASST) mouse/ MK16-Ras			Increases in suppressive myeloid cells, accelerates the ability of MDSCs to inhibit anti-tumor T cell responses [150]
Hepatocellular carcinoma			TAM recruitment [151]
Tumor-bearing mice (B16 melanoma, MC38 colon carcinoma, or EL4 lymphoma)			Regulates IL-4R expression on MDSCs thereby indirectly inhibiting the release of arginase (Arg1) [90]
Colorectal cancer	IL-17	Proinflammatory effect; promotion of congenital activity; activation of neutrophils and T-cells [152]	MDSC recruitment; more pronounced immunosuppressive activity of MDSCs; decrease in the number of CD8^+^ T cells; positive effect on Treg [153]
Esophageal squamous cell carcinoma (ESCC)			Correlation with CXCL2/CXCL3 ligands, enhanced tendency of inflammatory cells to migrate [75]
Breast cancer			Inhibits MDSC proliferation, promotes MDSC differentiation, reduces levels of TGF-β and IL-10 released by MDSCs and enhances the synthesis of pro-inflammatory factors [101]
Breast cancer (MCF-7)	IL-8	Proinflammatory effect; involved in lymphocytic infiltration in various cancers [149]	Cellular senescence phenotype [149]
Gastric cancer	TNF-α	Biological functions dependent on the type of activated receptors; possible proinflammatory and oncostatic effects [154]	Induction of PD-L1 expression on mast cells, indirect negative impact on T cell immunity [155]
Ehrlich’s ascites carcinoma (EAC) cells 4T1 mouse breast cancer cells			M1 TAM marker, prevents polarization towards the M2 subtype [64]
Lung cancer (NSCLC)	IL-33	Early inducer of inflammation [156]	Blockade of M2 TAM polarization, decreased recruitment of Tregs in TME; shaping functional immune surveillance [157]
N/A (TME imitating milieu)			Suppresses or enhances effector functions of cytotoxic/regulatory T cells, differentiation of CD8^+^ T cells, supports TCR-dependent activation of CD8^+^ lymphocytes/T lymphocytes [158]
Human lung cancer cell line/NSCLC (NCI-H1299 (ATCC® CRL-5803)	TGF-β	The predominant immunosuppressive activity; regulation of T lymphocyte activity; abolition of anti-tumor immune response [159]	Enhances the antiproliferative effect of MDSCs on T cells, Treg promotion through MDSCs, attenuated antitumor immunity [160]
N/A			TAN polarization to N2 subtype [67]
Esophageal squamous cell carcinoma (ESCC)			TGF-β-dependent Smad3 enhanced PD-1 expression on TILs in the TME [161]
Mouse model of pancreatic cancer (LSL-KRas^G12D^)	IL-1/IL-1R signaling	Strong proinflammatory effect; alternative action as a transcription factor [162, 163]	Senescence-associated secretory phenotype (SASP) [165]
Human mammary cancer-derived cells (MDA-MB-231, MCF-7)	Oncostatin M	Proinflammatory cytokine, induces endothelial activation [164]	Promotes M2 polarization via HIF-1α/ARG1/COX-2 [164]

## Proinflammatory and senescent phenotypes of cancer

Cancer-associated inflammation is a hallmark of many tumors, and often causes serious clinical outcomes. A systemic inflammatory response in colorectal cancer is of prognostic significance in terms of the neutrophil-lymphocyte ratio (NLR) in which preoperative a NLR ≥ 5 is correlated with a lower overall survival and a higher risk of a recurrence [166]. During inflammation, activated neutrophils release proteins and chromatin, which leads to the formation of a fibrillary matrix referred to as neutrophil extracellular trap (NET). This phenomenon appears to be dependent on the presence of certain cytokines, such as TNF-α, IL-6 and IL-8. It also causes hypercoagulability which, by disturbing the endothelial cell morphology that converts them to a procoagulant phenotype, may worsen a patient’s prognosis in more advanced clinical stages of cancer [167]. In organs where potential recurrences may occur, an inflammatory response seems to be predominant in the microenvironment that represents the premetastatic niche. It occurs by downregulation of selected tumor suppressors as well as by a positive influence on the glycolytic metabolism of cancer cells, which helps them to proliferate and to form metastatic foci [168]. Nearly half of the patients with metastatic colorectal cancer (mCRC) present significantly higher levels of at least two out of three inflammation markers, i.e., miR-21, IL-6 and/or IL-8. The inflammatory cancer phenotype is an unfavorable predictive factor for relapse-free and overall survival. In contrast, higher IL-6 concentrations have been found to exhibit independent prognostic value for overall survival in an unresectable cohort [169].

Transcription factors play a significant role in the release of pro-inflammatory factors. Zinc finger E-box binding protein 1 (ZEB1) and ZEB2 are transcription activators that have frequently been associated with cancer progression. The inflammatory breast cancer phenotype is, for example, partly regulated by ZEB1/ZEB2. ZEB1 enhances IL-6 release and activates STAT3, which results in an increased proliferation potential of cancer cells. Together with IL-6, both factors have been found to positively influence cancer-associated fibroblast proliferation [170].

Chromatin redistribution from the nucleus to the cytoplasm takes place during cellular senescence, which is associated with the regulation of an inflammatory response and the expression of certain oncogenes. Cytoplasmic chromatin is also abundant in cancer cells, and the expression profile of proinflammatory genes in some types of cancer cells is similar to that of senescent cells [171]. Cellular senescence may exert a suppressive effect on cancer or, conversely, lead to cancer progression. The senescence-associated secretory phenotype (SASP) is characterized by a set of biologically active mediators such as growth factors, cytokines, and extracellular vesicles that are secreted by aging cell populations. A negative implication of SASP is its potential promoting effect on tumor cell proliferation and metastasis. Since the biological significance of SASP depends on specific factors that are released, both immunosuppressive and proinflammatory effects may be exerted on immune responses, each with a different clinical effect [172]. SASP secretome activation in a mouse model correlated with a proinflammatory response and pancreatic cancer progression depending on the IL-1/IL-1R signaling pathway, which independently induced SASP gene expression and immune cell infiltration [165]. Apart from having a proinflammatory effect, paracrine release of SASP factors such as IL-6 and IL-8 may induce epithelial-mesenchyme transition (EMT) and lead to cancer cell invasion [173]. A proinflammatory secretory phenotype of ovary adenocarcinoma SKOV3 cells and a sub-population of cells aging during exposure to carboplatin (CPT) have been found to induce SASP, as evidenced by an increased expression of IL-1B and IL-8, among others. Supernatants from CPT-treated SKOV3 cells also enhanced STAT3 phosphorylation in human macrophages. Positive convergence of gene expression with EMT markers implies the potential importance of SASP in chemoresistance and cancer progression [174].

Senescent stromal cells promote local inflammation by increasing the release of proinflammatory cytokines, such as IL-6, as well as by redistributing cellular responses via increasing the CD45^+^ cell population, out of which a significant subpopulation shows a myeloid-cell immunophenotype. The senescent microenvironment induces inflammatory infiltration with a phenotype corresponding to a granulocytic and monocytic line of MDSCs, which is accompanied by a high co-expression of immunosuppressive factors, promotion of CD4^+^Foxp3^+^ T cells, increased IL-10 release and TGF-β and STAT3 expression. Fibroblasts may also exhibit an immunosuppressive influence in an aging microenvironment, since under such conditions they impair T cell responses and promote cancer cell growth. Some SASP proinflammatory cytokines, such as those secreted by aging fibroblasts and IL-6, also modulate the stroma in such a way that it has immunosuppressive properties, i.e., limiting the T cell response and promoting carcinogenesis [150]. Under some conditions, the potential effect of suppressing cell division by an aging cell population does not seem to affect transformed cells. In prostate cancer, SASP antiproliferative activity toward the cells surrounding an aging cell was observed only in healthy cells or aging cells with minor mutations and, importantly, had no such effect on tumor cells [175]. The suppressive effect of cellular senescence on systemic inflammation appears to be confirmed indirectly by an increase in release of IL-10 with age [176].

## Chronic inflammation-associated cancers - similarities and differences between chronic inflammation and tumors in modifying the immune response

The evolution over time of neoplastic transformation and subsequent malignancy engages immune responses and creates conditions corresponding to chronic inflammation, which is also an important characteristic of many inflammation-conditioned tumors [177]. Long-term exposure to antigens disturbs the metabolism of T cells, which may condition the effector functions of these cells at various stages of the chronic inflammatory process [178]. The vast majority of studies carried out so far do not assess this similarity and compare inflammatory markers between cancer and normal tissue, omitting the assessment of tissues affected by chronic inflammation, which may complicate the identification of significant factors relevant for cancer progression involved in neoplastic regulation of the host immune system [179]. In this section we compare the immunological parameters between chronic inflammation of specific organs and its corresponding cancers. Particular attention is dedicated to precancerous conditions developing in the course of long-term inflammation that significantly increase the risk of cancer initiation.

### Cirrhosis and hepatocellular carcinoma

During hepatic cirrhosis, thymopoiesis is disturbed, resulting in lymphopenia of Th cells expressed as a significant reduction in circulating naive CD31^+^ Th cells. It results from a disturbance of homeostasis between lymphocyte proliferation and mechanisms of their activation and apoptosis [180], induced by changes conditioned by organ dysfunction during chronic inflammation of the liver. Regulatory T cells (Tregs) and Th17 cells also seem to be significant. The Th17/Treg ratio is an important parameter of liver dysfunction in the course of chronic inflammation, and a risk factor for progression to hepatocellular carcinoma (HCC). Patients with a higher liver stiffness measurement (LSM) present significantly higher Th17 and lower Treg cell numbers compared to patients with a low LSM. The Th17/Treg ratio shows a positive correlation with LSM values, a high correlation with cirrhosis and appears to be a risk factor for HCC development in patients with hepatitis B virus (HBV) infection [181].

Chronic inflammation of the liver also predisposes to the occurrence of liver-resident immunoglobulin-A-producing (IgA^+^) cells that exhibit immunosuppressive activity through e.g. increased expression of IL-10 and the PD-L1 ligands. This hinders the antitumor effect of CD8^+^ T cells, which is expressed by a weakening of cytotoxic functions and a deterioration of tumor-associated antigen (TAA) detection [182]. In cirrhosis, the serum IL-10 level is elevated and the MHC class II expression on monocytes (CD14^+^) is reduced [183]. The immunosuppressive effect of chronic inflammation also seems to modify the systemic secretion of proteins that promote immune tolerance. During chronic HBV infection, Tim-3 serum levels have shown a significant correlation with the risk to develop HCC and, during malignant transformation, it was found to be an unfavorable prognostic factor [184]. Tim-3 belongs to a group of immunological checkpoint proteins, the expression of which mediates T cell depletion [185].

The vast majority of HCC cases results from chronic liver damage [186]. Immunological parameters important in carcinogenesis and in subsequent HCC development differ depending on the tumor-triggering factors – high values of Treg and CD8^+^ resident memory T cells have been observed in HBV-related HCCs, whereas in non-viral-related HCCs a significantly higher percentage of CD244^+^ NK cells has been observed, as well as CD8^+^ T cells expressing Tim-3. The mechanism of immunosuppression occurring in both types of HCC also appears to be different, where the mechanism associated with the induction of PD-1 ligand in Treg and CD8^+^ memory T cells remains more pronounced in HCCs with a viral etiology [187].

The intensity of inflammatory infiltration itself appears to have a differentiating value with regard to pathologic features. Inflammatory infiltration distinguishes the HCC microenvironment into three major immune subtypes, differing in the intensity of infiltration (high/mid/low), immunophenotype of immune cells, association with molecular classification of HCC and prognostic value, which depends more on the predominant components of infiltration rather than on local micro-spots of heterogeneous tumor tissue. The immune-high subtype is characterized by more pronounced tissue infiltration by B cells and plasmocytes, which are independent and beneficial prognostic factors.

The prognostic value of inflammatory infiltration in HCC also seems to be dependent on the individual histopathological subtype, where the immune-high subtype has a beneficial impact [188]. The activity of immunocompetent cells and their effector properties in HCC are linked with the stage of cancer. CD8^+^ T cells play a significant role in killing tumor cells by recognizing TAAs located on their surface, proteins with immunogenic potential originating from the host, conditioned by changes caused by mutation or anomalous expression. In HCC, the ability to recognize TAAs by CD8^+^ T cells is determined by the specificity of the epitope and appears to be dependent on the stage of cancer, since the highest ability of CD8^+^ T cells to respond to a specific TAA is observed in the early stages of cancer and is a favorable prognostic factor. The effector properties of CD8^+^ T cells against TAAs may explain the dominance of these cells in inflammatory infiltrations at the early stages of cancer [189]. B7 superfamily member 1 (B7S1/B7-H4/B7x/VTCN1) nullifies CD8^+^ T cell functions, and the expression of this molecule on myeloid cells in HCC is elevated, shows a positive correlation with CD8^+^ T cell dysfunction and promotes T lymphocyte depletion and co-expression with PD-1, which suggests a synergistic relationship between these two proteins [190].

The extent of the cytotoxic effects in this tumor type may also be affected by changes in humoral responses. In HCC, IL-7 expression is significantly reduced, and increases in this cytokine are associated with a better response to therapy, which probably results from the profitable effect of IL-7 on the cytotoxic functions of CD8^+^ T cells [191]. In contrast to cirrhosis, Tregs play an important role in HCC pathogenesis. The percentage of Tregs has been found to be significantly higher in patients with HCC than in patients with chronic inflammation associated with hepatitis C virus (HCV) and HBV infections [189]. Recruitment of Tregs to the tumor tissue may occur under the influence of the secretory activity of cancer cells and the specific actions of the secreted factors.

In HCC, Treg (FoxP3^+^) recruitment directed to inflammatory infiltration appears to be dependent on metalloproteinase-12 (MMP-12) overexpression, and it has been found that a significant positive correlation exists between both parameters [192]. The role of Tregs in cancer progression is also important in those arising due to chronic viremia. In HBV-associated HCC, the number of Tregs and Th17 lymphocytes has been found to be significantly higher in the peripheral blood than in the control group and the percentages of these cells have been found to be positively correlated with the stage of cancer and its size [193]. On the other hand, the level of Tregs in HCC is not always increased, but the activity of this cell population seems to be modified by the cancer.

Despite the possibility of undisturbed quantitative ratios of Tregs in HCC, their activity in cancer seems to be significantly higher. CD4^+^CD25^+^FoxP3^+^ cells isolated from patients diagnosed with HCC showed an increased transcriptional activity compared to controls, regardless the pathogenic basis of tumor development [194]. CD4^+^CD69^+^Foxp3^-^ Tregs present in HCC inflammatory infiltration suppress T lymphocytes through membrane-bound transforming growth factor-β (mTGF-β). The population of these cells in cancer outnumbers the cells with a CD4^+^CD25^+^Foxp3^+^ phenotype, they have the ability to release immunosuppressants such as IL10 and TGF-β1, and the number of these cells increases progressively in HCC along with the stage of the disease [195]. In a mouse model of HCC, both TGF-β and IL-10 have been found to be associated with tumor progression, and in vitro TGF-β has been found to promote the differentiation of Foxp3^+^CD4^+^ Tregs [196]. Under physiological conditions, Tregs determine immune system homeostasis by abolishing the effects of activation of the immune response through immunosuppressive impact determined by the synthesis of cytokines such as IL10 and TGF-β, and by effects on APCs and effector functions of T cells [197]. The secretory activity of cancer can, however, under certain conditions potentiate the immunosuppressive influence of these cytokines on the cellular responses.

Tregs normally express the GARP receptor encoded by the *Lrrc32* gene, which under pathological conditions may increase the oncogenic potential of the tumor and increase the biological activity of TGF-β, and its overexpression induced by cancer may lead to Treg activation [198]. Promotion of immunosuppression in HCC may also occur through the bioactivity of fibroblasts present in the tumor tissue, which through IL-6 induce activation of STAT3 in neutrophils, which simultaneously results in a positive co-expression of PD-L1 ligand within these cells and, by doing so, may reduce effector T cell activity against tumors [199]. STAT3 signaling also appears to show a relationship with an aggressive course of cancer. Polarization of macrophages into an unfavorable M2 subtype has been found to be associated with epithelial-mesenchymal transition in HCC cells in which the TLR4/STAT3 pathway appears to be involved [200].

### Chronic pancreatitis and pancreatic cancer

In the inflammatory infiltration in chronic pancreatitis (CP) patients, Th1 and Th17 cells predominate [201]. The *Bach2* gene is repressed in the course of chronic pancreatitis and is dependent on T cell polarization towards the Th17 type. Despite the higher number of CD4^+^ T cells in CP than in controls, the Bach2^+^CD4^+^ T cell number was found to be lower and the rs9111-TT gene variant was found to be dependent on the stage of inflammation, expressed by the morphological parameters of the organ [202]. The T cell type 1 response appears to have similar traits in both CP and cancer, but in chronic inflammation Treg cells show a stronger response to some antigens expressed by increased IL10 secretion, which is accompanied by an increased concentration of this immunosuppressive cytokine in the inflamed tissue together with reduced IFN-γ, compared to the levels in cancer. The population of cells with the CD3^+^CD4^+^CD25^+^FOXP3^+^ phenotype was also more numerous in CP than in the normal control [203]. The level of expression of certain immunosuppressive factors may vary depending on the etiology of chronic inflammation. Expression of TGF-β in the histochemical evaluation was found to be explicitly weaker in autoimmune chronic pancreatitis than in chronic inflammation resulting from alcoholism [204].

In pancreatic ductal adenocarcinoma (PDAC), CTLA-4^+^ Tregs infiltrate tumor tissue relatively early and these cells tend to be predominantly redistributed to lymph nodes surrounding the tumor, which is associated with progression of the disease. CTLA-4^+^ Tregs also regulate neoplastic inflammatory infiltration by CD4^+^ T cells through interaction of CTLA-4 with CD80, which leads to a reduction in the number of CD4^+^ tumor-infiltrating T cells, preventing CTLA-4 from interacting with CD80 in an animal model of tumor infiltration by CD4^+^ lymphocytes [205]. Treg cells in the animal model of pancreatic cancer were found to be associated with tumor progression and to program dendritic cells (DCs) to abolish antitumor activity. Tregs integrate with CD11^+^ DCs and suppress the expression of ligands responsible for the activation of CD8^+^ T cells [206].

In the pathogenesis of pancreatic cancer (PC), cytotoxic lymphocytes are also important. CD8^+^ cells are a favorable prognostic parameter in pancreatic cancer [207]. In PDAC, activation of JAK2/STAT3 cell signaling with the participation of REG3G was found to induce immunosuppression by limiting the antigenicity of tumor cells, suppressing CD8^+^ cell function, causing variable expression of Th2 cytokines and increasing the proliferation of tumor cells [208]. In pancreatic cancer, CD25^+^CCR6^+^ Th17 cells showed a stronger suppressive effect than the CD25^-^CCR6^-^ Th17 phenotype. This phenotype, after stimulation, showed a more pronounced expression of CTLA4, and incubation of these cells with CD8^+^ T cells showed a more pronounced antiproliferative effect on CD8^+^ T cells. Moreover, the CTLA4^+^ Th17 cell number was found to be higher in TILs than in controls [209]. In addition, tumor cells in pancreatic cancer tend to release IL-10, which induces an immunosuppressive effect by limiting the activity of T-lymphocytes [210].

### Inflammatory bowel diseases and colorectal cancer

In inflammatory bowel disease (IBD), the expression of CD39 in the FoxP3^+^ Treg immunophenotype is reduced, whereas an increase in CD39 expression is associated with a positive response to treatment. CD39 expression in Tregs present in the peripheral blood has been found to serve as a marker of remission of clinical signs of the disease [211]. In this group of diseases, the percentage of plasmocytoid DCs and myeloid DCs in blood is also lower compared to that in normal controls, and in ulcerative colitis (UC) the expression of the CD200 ligand on plasmocytoid DCs has been found to be increased compared to that in controls. Conversely, Th17 cells show an inverse relationship [212]. In patients with UC, the number of Tregs (CD4^+^CD25^+^CD127^low^FoxP3^+^) has been found to be lower than that in normal controls, and the suppressive activity of this lymphocyte subpopulation has been found to be inversely correlated with the clinical stage of the disease [213]. In the course of UC, decreased levels of immunosuppressive cytokines, such as IL-10 and IL-35, have been found. Their concentration was found to be dependent on the expression of its corresponding microRNAs in Tregs, i.e., decreased levels of IL-10 and IL-35 in Treg cultures from patients with UC correlated with decreased miR-21/miR-146a/miR-155 levels and an increased miR-31 level [214].

Immunoprofiling of patients diagnosed with UC revealed a time-qualitative differentiation in phases of chronic inflammation and an increased expression of relevant antigens. In the acute phase of the process, CD11b^+^ and CD64^+^CD14^+^CCR2^+^ macrophages appeared as well as monocytes, with an additional expression of TARC and HGF, while in the “remodeling condition”, NKT cells and monocytes expressing TSLP and TGFβ1 receptors were found to be present. The TARC and TGFβ1 receptors seem to differentiate between these two chronic inflammatory conditions [215]. In IBD, TGF-β-activated kinase 1 (TAK1), which is a known inflammatory regulator, is overactivated and its inhibition suppresses the release of proinflammatory cytokines and reduces inflammatory infiltration and clinical symptoms of intestinal disease. With regard to UC and CD, activation of TAK1 at an early stage of the disease usually occurs in intestinal epithelial cells, whereas in later stages of the disease hyperactivation of this kinase occurs in non-epithelial cells, mainly macrophages [216]. The level of expression of proinflammatory IL-17A and its receptor IL-17RA has been found to be significantly higher in UC and in benign gut polyps than in CRC. The levels of other ligands from the IL-17 family (E/F) and IL-17RB receptors, along with neutrophil and mast cell infiltration, were significantly reduced in cancer compared to benign lesions, unlike the IL17RC ligand, which together with CD3^+^ cell infiltration was more highly expressed in CRC than in polyps [217]. The levels of other cytokines may also change. Analyses of biopsies have shown that cytokines IL-19 and IL-20 are overexpressed in the active phase of inflammatory bowel disease [218].

A positive correlation between Treg cells and tumor associated macrophages (TAMs) has been found in CRC. Both cell populations have shown an unfavorable prognostic value associated with a significantly increased risk of recurrence and death, whereas Tregs demonstrated a significantly higher predictive value in predicting recurrence than TAMs [219]. Tregs also limit the migration of T cells into tumor tissues by lowering CXCL10 release by endothelial cells and inhibiting CXCR3-mediated signaling. CRC-associated vascular endothelial cells tend to overexpress CXCL9 and CXCL10, both ligands for the CXCR3 chemokine receptor located on Th1 cells, whose activation positively regulates the degree of inflammatory infiltration. The sources of the aforementioned ligands may also be other immunologically competent cells such as T lymphocytes, B cells and monocytes [220]. Differences in the level of expression of these chemokines, depending on Tregs, may be the cause of varying TIL levels in cancers. With respect to the tumor microenvironment, the number of Tregs has been found to be much higher than that in normal controls. In addition, PD-1/CTLA-4 and PD-1/CD39 are co-expressed in these cells, which may suggest a synergistic effect of these markers in the induction of suppression of the immune response. Despite the comparable number of CD4^+^ T cells in inflammatory infiltration in normal and tumor tissues, a higher co-expression of PD-1 and CTLA-4 has been found in the T lymphocyte pool present in tumor tissues compared to control tissues [221].

**Fig. 2:**
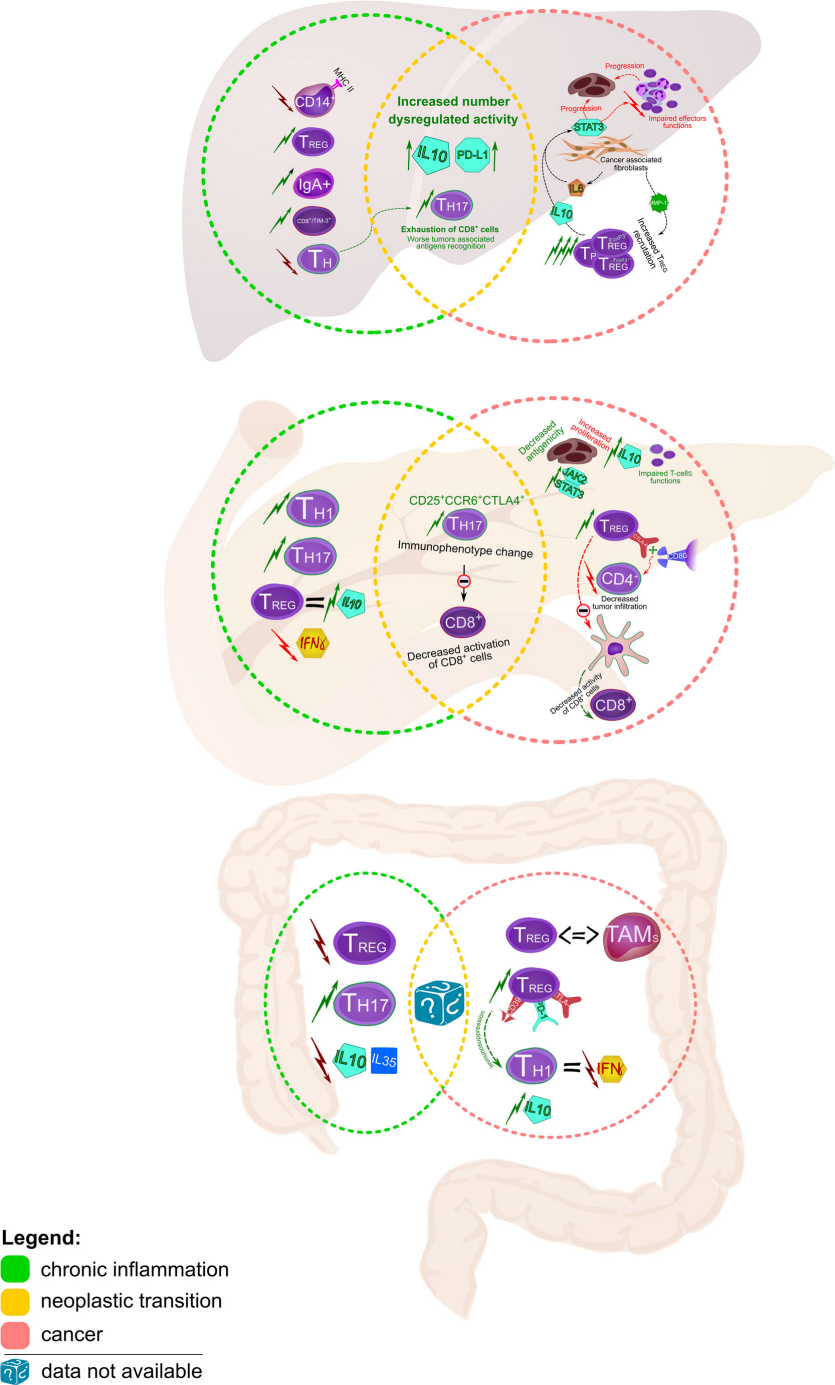
**Chronic inflammation may predispose patients to the development of neoplasms.** Long term activation of inflammatory cells leads to changes in their immunophenotype, secretory activity, function and subpopulation numbers. Discrete molecular changes in the phenotype of lymphocytes and their ongoing activation in inflammatory sites are the cause of functional exhaustion of these cells, which may lead to immune suppression and, consequently, increase the chance of neoplastic transformation

The neoplastic-induced state of hypoxia is associated with immune suppression expressed as, e.g., varying regulation of CD4^+^ and Treg cells. In a mouse model with induced colitis-associated colon cancer, an increase in Treg immunosuppressive activity has been observed. This increase was accompanied by a decreased proliferation and IFN-γ synthesis by Th1 lymphocytes. Supernatants obtained from biopsy material also exhibited a significantly higher level of IL-10 in cancer patients than in normal controls [222]. The relations of the processes discussed above are presented in a broader context in Figure 2.

## Postoperative changes in immune responses suggest an immunosuppressive effect of cancer cells

### Wound healing

Surgical wounds change the parameters of hemodynamic, hormonal and metabolic homeostasis as well as the systemic inflammatory response in which the released cytokines play a particularly important role. During the first 36 hours after a surgical incision, inflammation is activated in the form of increased involvement of immune cells, increased expression of adhesion molecules and increased synthesis of pro-inflammatory cytokines, such as IL-1, IL-6, IL-8 and TNF-α. In the second phase of this process, lasting approximately 2 days, there is a decrease in inflammatory activity through immunosuppression, with participation of among others IL-10 and IL-1RA, which regulate both humoral and cellular responses [223]. Surgical traumatization of tissue inducing acute inflammation leads to a cascade of regenerating processes. In the initial phase of wound healing (1-3 days), macrophages play a major role. They regulate the expression of inflammatory genes in a mechanism dependent on Notch signaling. Reduced activation of this pathway leads to a subsequent reduction in expression of some proinflammatory cytokines, such as IL-1B and TNF-α [224]. Induction of acute inflammation in an animal model also caused a progressive increase in IL-2, IL-6 and C-reactive protein (CRP), which appeared in the tested sera in a specific sequence [225]. IL6 levels are usually elevated up to 3 days after surgery and normalized on day 7 after surgery. Interestingly, IL-6 returns to normal levels faster than CRP [226]. It should be kept in mind that the abovementioned timeframe can be regulated by numerous external factors. One of them is a different percentage of postoperative complications that is strictly dependent on the individual skills and proficiency of the surgeon [227]. Another one, difficult to clearly determine/predict, is the profile of selected markers of inflammation, which may vary depending on the molecular structure of the protein, the primary location of local inflammation and the patient's state of health. For example, CRP is one of the acute phase proteins whose overexpression occurs during activation of the immune response, and the source of production of this inflammatory marker may not only be hepatocytes, but also other cell types such as endothelial cells, adipocytes, smooth muscle cells and immune cells. Some CRP isoforms may specialize in performing anti-inflammatory functions, while others may induce the secretion of cytokines and chemokines that promote pathological processes associated with the regulation of angiogenesis and cell proliferation. CRP isoforms also show tissue-dependent, specific biological activities [228]. The CRP protein also has the ability to regulate the expression of various genes, including those encoding proteins involved in immunological functions such as CD59, whose mRNA level was found to be elevated in peripheral blood induced by CRP [229]. Some CD antigens determine the immunophenotype of inflammatory cells, and they also perform functions in the regeneration of specific organs.

The CD46 receptor located on the surface of epithelial cells may tighten the epithelial barrier, which may also be important in the wound healing process. Activation of CD46 accelerates this process and regulates the formation of adhesion molecules, such as E-cadherins, that form intercellular connections. SPAK and alpha-E-catenin signaling are likely to be involved in this process. Signaling induced by CD46 activation may also regulate the Notch pathway, affecting not only the integrity of epithelial cells [230] but also immune function regulation. In the case of some chronic autoimmune diseases, the cofactor CD46 plays a significant role in regulation of the complement system and its synthesis has been found to be positively regulated by CRP [231, 232].

In the process of regeneration of damaged skin, particular subpopulations of immunologically competent cells play a significant role. Wound healing is accompanied by, among others, dendritic epidermal T cells, which induce IGF-1-mediated local inflammation and promote epithelial cell proliferation. Delayed scar formation may be caused by the regulation of cytokine expression (IL-1B, IL-23, IL-17A) released by Vy4 T cells activated through the T cell receptor (TCR) pathway [233]. Invariant natural killer T (iNKT) cells, which have antigenic features of both T cells and NK cells, regulate neutrophil apoptosis by the expression of IFN-γ, which limits the damage caused by the release of neutrophil elastase, which in turn accelerates the wound healing process [234]. The wound healing process is disturbed in neoplastic diseases, where delayed regeneration actions have been observed at every wound healing stage, and monocyte and T cell infiltration were found to be significantly less in cancer cases than in normal controls, which was associated with a local limitation of neovascularization and collagen synthesis [235]. Although delayed regenerative processes of normal tissues during active carcinogenesis show signs of tumor-induced suppression of immune responses, they may also be accompanied by tumor progression.

In studies carried out on an animal model, it has been found that postoperative fluids derived from sarcoma patients have a pro-tumor effect in terms of angiogenesis stimulation and promotion of cellular proliferation, including the regulation of adhesive junctions [236]. Surgical procedures in oncological patients also involve systemic immune responses, but it should be remembered that neoplastic processes are usually associated with a preceding induction of chronic inflammation and, hence, changes at the molecular level may assume different kinetic and qualitative values. During radical resection of gastric cancer, an intraoperative activation of inflammation parameters in both adipose tissue and blood serum has been observed, i.e., the expression of IL-6, CC-chemokine ligand-2 and IL-1β was increased in peritoneal adipose tissue. The IL-6 level was also significantly elevated in the serum, and persisted up to one week after resection [237].

The surgical procedure may induce changes in humoral responses. During surgical traumatization of tissues also changes in cellular immunity may occur. Shortly after resection of gastric cancer, an increase in neutrophils accompanied by a decrease in the number of dendritic cells and T cells has been observed [237], which may suggest tumor-induced suppression of antigen presentation and impairment of effector functions. Postoperative cellular stress in people without a malignant tumor usually activates an immune response and mobilizes particular T cell subpopulations, which is associated with a local inflammatory infiltration of these cells [238]. On the other hand, surgical intervention normally triggers a short-term immunosuppressive effect associated with the wound healing process, which may have an unfavorable prognostic value for patients with cancer due to an increase in the risk of metastases. Local immunotherapy in the perioperative period, which increases among others the percentage of T cells and dendritic cells, may result in beneficial prognostic effects [239]. However, the surgical state of immunosuppression seems to be independent of the healing process of the tissue itself, but instead to show a correlation with tumor grade. Surgical resection of benign tumors with an epithelium-mesenchymal framework does not lead to postoperative immunosuppression, and proinflammatory markers remain at a similar level after surgical resection and before decompressive surgery [240].

Wound fluid (WF) is a rich source of cytokines modifying the functions of immune responses which have been shown to promote the progression of cancer in vitro. The rich content of cancer cells with a stem cell phenotype in WF is particularly interesting. In breast cancer, this phenotype is presumably induced by activation of STAT3, which strongly predicts the presence of WF [241]. In women with breast cancer who underwent surgical organ reconstruction after mastectomy, the cytokine profile assessed on the day of surgery and two days later was diverse, i.e., the levels of some immunosuppressive cytokines were higher at the beginning, and subsequently decreased significantly. It should be emphasized that in case of some cytokines evaluated in the WF, their concentration was significantly influenced by the level of stress of the examined patients [242]. Increased stress levels reduce the number of macrophages (CD68^+^), leukocytes (CD45^+^) and Langerhans cells (CD1a^+^) and significantly limit the activity of immune cells in the skin by regulating the expression of HLA antigens, which is associated with impaired wound healing. It should be noted that also age plays a significant role in modulation of the immune response to stress and that it determines quantitative parameters of some immunologically competent cells [243]. Finally, the postoperative immune response is known to be modified by several somatic factors that may contribute to changes in cellular metabolism. In obese patients with a metabolic syndrome who underwent surgical resection of esophageal adenocarcinoma, the CRP rate was found to be higher than that in normal controls, and a higher ratio of CRP/albumin was also observed one and two weeks after surgery [244]. Interestingly, CRP can also perform biological functions in small concentrations and exhibit suppressive effects against acquired immunity, and disturb the maturation and functions of dendritic cells, which promotes T cell tolerance [245]. Nevertheless, the level of CRP seems to have no prognostic value. In oncological patients with CRC metastases to the liver who underwent surgical treatment, the postoperative range of CRP concentrations did not affect survival and was no good prognostic marker [246]. STAT3, which promotes cell proliferation of cancer cells, is one of the proteins that is activated in cellular signaling of immunosuppressive cytokines [33, 247].

### Surgical resection changes the immunophenotype of tumor tissue

In epithelial ovarian cancer, similar parameters of CD8^+^ T cells have been found in primary and recurrent tumors. However, the number of Tregs was significantly more pronounced in recurrences and showed a positive relationship with relapse-free survival [248]. In serous ovarian carcinoma, some differences between primary and recurrent tumors have been noted, i.e., a relatively higher expression of CD4 and MHC1 in recurrent tumors. Interestingly, higher CD3 levels in recurrences have shown a relationship with response to platinum-based chemotherapy [249].

In some types of neoplasms primary tumors also seem to be differentiable from relapses when considering macrophages. In gastric cancer, for example, CD163^+^-polarized TAM infiltrations showed a positive relationship with a more aggressive course of disease and with cancer stem cell (CSC) markers, and their higher numbers in recurrences served as an unfavorable prognostic factor [250]. The central nervous system is usually isolated from the systemic immune response. Concordantly, in glioblastomas no differences in selected immunological markers between primary tumors and relapses have been noted [251]. Although the spectrum of immunological features of primary gliomas and recurrent tumors appears to be similar, secondary tumors have shown a restricted TCR repertoire clonality and a greater activation of memory T cells [252]. The conclusions on this issue are not unequivocal. Sabrina Heynckes et al. described significant differences in PD-L1 expression between primary and recurrent glioblastomas, where PD-L1 expression levels were higher in de novo tumors than in relapses at both the protein and mRNA levels. The authors argue that it explains the relatively worse response of secondary tumors to immunotherapy, where PD-L1 is one of the targets [253].

### The postoperative period is accompanied by tumor-induced immunosuppression

Surgical procedures performed on healthy animal models have led to an increased expression of inflammatory markers in the postoperative period [254]. This is different in many solid tumors. After surgical removal of cancer, cancer cells that remain in the body show immunosuppressive activity, which leads to an increase in immunological dysfunction [255]. Regulatory T cells with immunosuppressive properties in patients with advanced ovarian cancer exhibited higher values than in normal controls and in tumors described as benign. Moreover, their number was significantly higher before surgery than after surgery. Interestingly, a gradual increase in this lymphocyte population has been observed one week after surgical resection of the tumor [256].

**Fig. 3:**
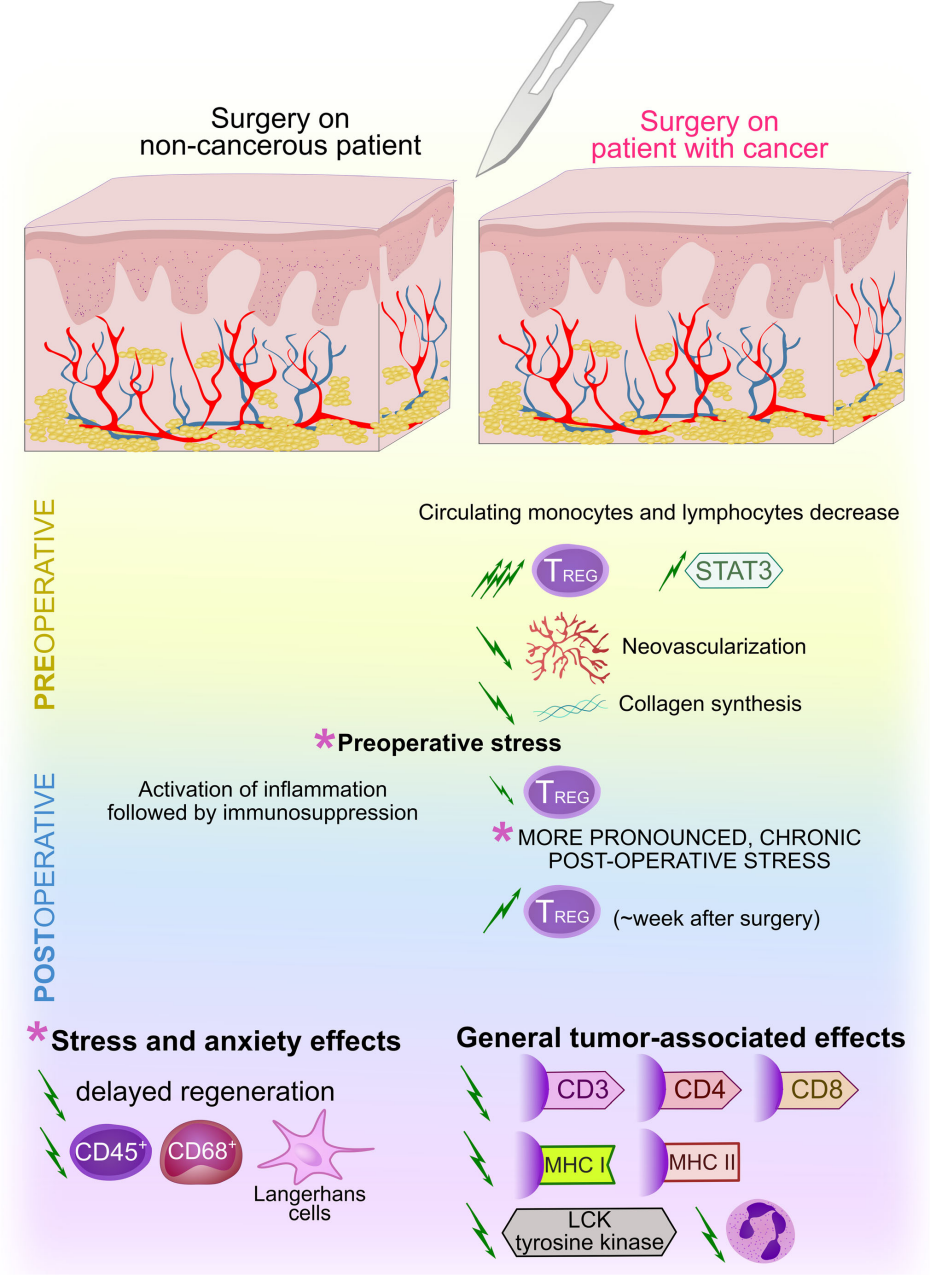
**Cancer-associated immune suppression delays wound healing after tissue injury sustained during oncologic resection.** Surgery does not always allow complete removal of the neoplasm and remnant cancer cells may aggravate immune suppression. Inherent characteristics of cancer patients may indirectly suppress the immune system – some psychological factors such as chronic stress may enhance this process. Surgery modulating immune activity has impact in a specific way. Immune responses to surgery in oncologic and nononcologic patients are compared chronologically

Postoperative changes in the expression of immunoregulating genes in peripheral blood seem to confirm the adverse effect of the tumor on the functions of T cells and NK cells, leading to impaired signal transduction, reduced APC cell capability and variable cytokine co-expression [255]. NK cells have a suppressive effect on the neoplastic process, and their reduced number may promote metastasis and proliferation of the tumor [257]. In solid tumors, CD8^+^ T cells are often dysfunctional, which probably occurs at an early stage of tumorigenesis, and the molecular profile of these cells resembles that of dysfunctional T cells at late stages of the disease [258]. In the postoperative period genes encoding MHC I and MHC II, TCR (CD3, CD4, CD8) and lck tyrosine kinase are downregulated. The last gene activates ZAP-70, which is a key protein in cellular signaling and is important in T cell activation and overexpression of immunosuppressing cytokines, such as IL-10 [255]. From a more practical perspective, the resulting tumor-induced immune deficits may increase the incidence and severity of secondary complications. A serious problem in patients treated surgically for lung cancer is postoperative infections, of which significant risk factors include patient age and cancer stage [259]. In tumors well isolated from the systemic inflammatory response (e.g. central nervous system tumors), the incidence of postoperative infections, including severe life-threatening complications such as sepsis, is much higher than that in classical debulking surgery conditioned by trauma or degeneration [260, 261]. The described relationships may be dependent on a decrease in cellular responses that occur in neoplastic processes. In patients with bone cancer a postoperative, progressive decrease in the number of neutrophils and the activity of these cells has been observed [262]. In non-small cell lung cancer after lobectomy, the ratio of neutrophils to leukocytes in peripheral blood has been found to be a favorable prognostic parameter [263]. Figure 3 highlights the relationships between the processes described above (Fig. 3).

## Metabolic alterations conditioned by cancer cells and their impact on tumor immunity

### Lipids

Increasing the energy demand of a developing cancer entails provision of the right number of substrates that are necessary for proliferation and further tumor growth. Metabolic reprogramming varies significantly between cancer and normal cells. During oncogenesis glucose metabolism often intensifies, as well as the synthesis of amino acids and lipids, which results from genetic changes occurring in transformed cells. These ongoing changes may modify the parameters of the tumor microenvironment and cause it to progress [264]. Human triple-negative MDA-MB-231 breast cancer cells actively capture fatty acids from the extracellular environment, which aggregate in the cells in the form of intracellular lipid drops (LDs). Tumor cells treated with medium obtained from patients with a high body mass index (BMI) containing increased concentrations of non-esterified fatty acids (NEFAs) tend to exhibit significantly higher PLIN2 expression levels associated with LD release and formation, which suggests that adipose tissue can regulate lipid metabolism in cancer cells [265]. Adipose tissue is important for regulating immune responses in individuals with visceral obesity. However, the activity of adipocytes was found to be strictly dependent on the appropriate tissue compartment and the surface area of the fat tissue studied in vitro. Among patients diagnosed with CRC, the transcriptional activity of visceral adipose tissue (VAT)-associated genes was found to be different from that of subcutaneous adipose tissue (SAT). The VAT transcriptome was characterized by increased expression of proinflammatory cytokines such as IL-6 and IL-8, as well as some adipokines. A higher fat content in both cases revealed proinflammatory activity and impaired lipid metabolism [266]. The importance of VAT in the pathogenesis of CRC was not only related to dysregulation of metabolism and changes in inflammatory parameters, but also to proangiogenic effects [267].

In one of the proposed models for assessing the clinical importance of mutations related to the progression and metastasis of breast cancer, mutations associated with fatty acid metabolism showed significant relevance. One of the immunosuppressive cytokines, TGF-β, may lead to distant bone metastasis and is found in higher concentrations in connective tissues [268]. The triggers that promote suppression of the immune response seem to be directly dependent on the synthesis of individual lipid groups under certain conditions. The anti-inflammatory phenotype of CD4^+^ T cells has been found to be associated with cholesterol biosynthesis, which leads to release of IL-10 by human Th1 lymphocytes. The cholesterol biosynthesis pathway appears to regulate c-Maf expression in T cells, which in turn controls the expression of IL-10. The impaired cholesterol biosynthesis pathway was found to affect c-Maf in a similar manner as IL-10 release [269].

Lipids are not only an important energy resource for cancer cells but can also induce carcinogenesis. Impaired lipid metabolism, characteristic of some glycogen storage diseases, has been found to be associated with a higher risk of cancer development. In glycogenosis, the risk of HCC was found to be increased by a high-calory diet, which reprograms the metabolism of hepatocytes in a direction resembling that found in cancer. Conditions leading to cell proliferation resulting from increased glycolysis and increased fatty acid accumulation disrupt cellular defense abilities, including the regulation of catabolic processes [270]. Some metabolic disorders appear to be associated with the induction of early stage carcinogenesis. Elevated HbA1c levels, abdominal obesity and hypertension correlate with the risk to develop gastrointestinal polyps. In addition, improper lipid metabolism with a high triglyceride to HDL ratio has been found to increase the risk to develop serrated adenomas [271].

### Cell cycle phases change the activity of inflammation-associated enzymes by regulating the metabolism of amino acids

Metabolic changes in transformed cells can disrupt the cell cycle. Intense arginine uptake during the S/G2M phase noted in tumor cells leads to the release of ornithine, which has been associated with mitochondrial arginase 2 (Arg2) activity. This has not been observed in normal epithelial cells [272]. In breast cancer, arginase and nitric oxide synthase (NOS) seem to be important for disease progression. The activity of arginase varies at different stages of cancer development, and the measurable level of the enzyme in sera also changes after chemotherapy. The level of arginase activity has been found to increase with the clinical stage of the disease, whereas after pharmacotherapy it approached the level of that in the control group. Nitrite anion levels were also found to be different between stage II breast cancer cases and controls, and they decreased after chemotherapy [273].

### Hypoxia and glycolysis

Some of the kinases that regulate glycolysis in cancer cells may also influence the immune system. High expression of type Iγ phosphatidylinositol phosphate kinase (PIPKIγ) is more frequently observed in CRCs than in normal tissues and is associated with a poor clinical outcome. Upregulation of PIPKIγ in colorectal adenocarcinomas enhances their glycolysis and proliferative activity, whereas PIPKIγ knockdown has been shown to be associated with inhibition of the PI3K/Akt/mTOR/c-Myc-HIF1α signaling pathway [274]. PIPKIγ may also be an important factor in the regulation of inflammatory infiltration. In an animal model it has been found that PIPKIγ activity increases the production of some phospholipids of cell membranes such as PtdIns (4,5)P2 involved in the recruitment of neutrophils [275]. In relation to cancer, this kinase also regulates the secretion of suppressive factors. In triple negative breast cancer cells, PD-L1 expression was found to be dependent on PIPKIγ through a mechanism mediated by activation of NF-κB [276].

Some metabolic disorders also change the molecular profile of MDSCs. Tumor-derived MDSCs show higher suppressive activity than controls, which was also found to be accompanied by a higher rate of glycolysis. The expression levels of glycolysis-associated genes, such as *Glut1*, *Hk2*, *Gpi*, *Tpi*, *Eno1*, *Pkm2*, *Lhda* and *Mct4*, were found to be significantly higher in tumor-MDSCs. Inhibition of glycolysis results in disruption of the suppressive potential of MDSCs. This effect was found to be regulated by the mammalian target of rapamycin (mTOR), whose level of phosphorylation is significantly higher in tumor-derived MDSCs [277].

The tumor microenvironment (TME) plays an important role in modifying immune parameters. An hypoxic TME has been associated with the expansion of MDSCs and the upregulation of expression of PD-L1, which contributed to the suppression of antitumor T cell immunity [278]. Acidification of the TME and glycolysis of tumor cells have a significant impact on the regulation of the immune response. Tumors that developed under conditions of a highly acidic microenvironment have been characterized by a high glycolytic activity regulating the expression of ICER in TAMs, which in a cAMP-dependent manner limited the effectiveness of the antitumor immune response [279]. Inflammatory cells exhibit variable expression levels of inflammatory factors in acidic environments. Polarization of M1 macrophages to M2 in a hypoxic environment has been associated with a downregulation of complement component 9 (C9). Its high expression in macrophages was found to be a beneficial prognostic factor in human NSCLC [280]. M2 polarization has been found to be associated with hypoxia-based upregulation of Nrp-1 expression, which also contributed to an increased recruitment of macrophages in vivo [281]. The immune cells themselves also appeared to be involved in inducing the above processes, which modified the expression of the corresponding cytokines. TAMs intensified hypoxia as well as oxygen glycolysis in non-small cell lung cancer (NSCLC) via AMP-activated protein kinase and peroxisome proliferator-activated receptor gamma coactivator 1-alpha, and the secretion of TNF-α [282].

### Tumor-induced metabolic changes interfere with the profile of immunosuppressive cytokines

Variable cholesterol levels in certain T cell subpopulations may lead to impaired expression of specific cytokines, such as IL-9, which affects the differentiation of immune cells and causes a change in the antitumor response in vivo [283]. Patients with breast cancer have been found to present a significantly reduced total level of cholesterol, triglycerides, high-density lipoproteins (HDL) and low-density lipoprotein (LDL) cholesterol compared to healthy controls [284]. The level of some lipoproteins also seems to be of prognostic significance. In case of CRC, for example, elevated high-density lipoprotein cholesterol (HDL-C) levels during chemotherapy showed a positive effect on overall survival (OS) and disease-free survival (DFS) [285], whereas the higher level of LDL-C found in patients with metastatic CRC (mCRC), as well as the LDL-C to HDL-C ratio (LHR), acted as independent indicators associated with a worse prognosis [286].

HDL stimulates the release of cytokines, including IL-10 [287]. In turn, IL-10 itself affects lipid metabolism, which leads to severe dyslipidemia, low HDL-C, low LDL-C, and elevated triglyceride levels [288]. A study on the anti-inflammatory effect of HDL-C in animal models revealed that this group of lipoproteins inhibits activation of NF-κB, MAPK and ERK [289]. NF-κB can induce NFATc1/αA in lymphocytes [290]. Inactivation of NFATc1, activated by TCRs in T cells, limits the effector functions of lymphocytes as well as a range of cytotoxic responses [34].

Metabolic changes in the TME can induce oxidative stress and subsequent Treg apoptosis, during which this subpopulation of lymphocytes releases ATP into the extracellular space. With the participation of CD39 and CD73 ecto-nucleotidases, ATP is converted into adenosine, which shows immunosuppressive activity as a result of interaction with the A2A receptor [291]. Hypoxia in the TME enhances the expression of CD39 and CD73, resulting in the accumulation of adenosine in the extracellular environment, which activates A2R receptors. Activation of A2aR and A2bR receptors on T cells leads to inhibition of effector functions of lymphocytes and prevents activation of the TCR. The presence of A2aR/A2bR receptors induces tumor suppression via CD8^+^ T cells through the A2aR receptor [292].

Cancer-induced hypoxia changes the expression of T cell genes, which may lead to resistance to chemotherapy [293]. Secretory properties of Tregs in gastric cancer revealed a suppressive potential of these cells as a result of a strong overexpression of IL10 and a less pronounced TGF-β synthesis [294]. TGF-β is a cytokine that induces the Foxp3^+^ phenotype in lymphocytes [295], while IL10 is an immunosuppressive factor whose increased concentration has been observed in chronic inflammation as well as in cancer [296, 297]. With regard to chronic HCV infection, some polymorphisms within the *IL10* gene have been associated with progression towards HCC [298]. The IL10/IL10R cell signaling pathway promotes macrophage polarization to the M2 phenotype [299].

## Extracellular vesicles modify the immune response

Exocytosis is a complex phenomenon of intercellular communication that occurs commonly in living organisms, the result of which is that molecules are transported through vesicles surrounded by a bilayer of lipids [300]. The ability of vesicles to transport various membrane and cytoplasmic proteins and other regulatory factors, including nucleic acids, suggests mediation in biologically important functions at the molecular level [301]. An important role of exocytosis is also seen in neoplastic processes. A prominent role during the initiation and progression of cancer is currently attributed to exosomes [302, 303].

### Exomeres

Methodological advances have enabled an accurate differentiation of exosome populations into exomeres (with a diameter not exceeding 50 nm), small exosomes (Exo-S, 60-80 nm diameter) and large exosomes (Exo-L, 90-120 nm diameter) [304]. Each of these subpopulations may show qualitative and functional diversity, depending on the source of origin, the diagnosis and the patient’s condition. The biological role of exomeres in the development of human diseases has not yet been determined, but these vesicles have the ability to transport biologically active factors that regulate cell metabolism and activity [305]. Exomeres secreted by tumor cells not only show morphological differences compared to other exosomal subpopulations, but also differ from Exo-S and Exo-L proteomic profiles in terms of the conveyed cargo. These vesicles contain proteins that regulate metabolism, glycolysis and hypoxia [306], which may be important in the pathogenesis of cancer and in the regulation of tumor immunity.

### Small and large exosomes

Due to the small size of these vesicles, the detection of exosomes is hindered by conventional methods and requires the use of appropriate techniques, which also have some limitations [307]. Morphological parameters of exosomes have not been strictly defined, and the range of their diameter is differently determined. Minciacchi et al. estimated the diameter of exosomes in the range of 30-100 nm [301], while Kalluri suggested a 40-150 nm range [308]. An interesting relationship was noticed by Thery et al. who, based on research conducted by Raposo et al., noted that the diameter of exosomes may depend on the type of cells from which they were released. B cells secreted the most homogeneous population which fell in the 60-80 nm range [309, 310]. According to Zhang H. et al., the differentiation of exosomes is more complicated. Hence, new subpopulations of vesicles may still be discovered [304, 306].

Many cell types can release exosomes, including inflammatory and cancer cells [308, 309]. The content of exosomes has shown the presence of proteins associated with antigen presentation, as well as factors corresponding to IL-2/STAT5 signaling [306, 309]. Inflammatory cells, by releasing exosomes, can condition the functioning of other cells. An example is Tregs, which by transporting vesicles containing microRNAs (miRNAs), regulate the proliferation, activity and secretory function of other T cells [311]. Cancer cells may also regulate inflammatory cell functions through extracellular transport. Gastric cancer-secreted exosomes, for example, through transporting miRNAs, regulate the expansion and activation of MDSCs [312]. In addition, oral squamous cell carcinoma (OSCC)-tumor-derived exosomes (TEXs) have been reported to affect γδ T cell expansion and cytotoxicity [313]. Hypoxia and the oxygen level also affect exosome activity. Hypoxic exosomes derived from MDSCs have shown a suppressive effect on γδ T cells as opposed to normoxic exosomes [313]. MDSC-derived exosomes exhibit quantitative RNA and protein profiles distinct from parental MDSCs, which suggests that exosomes may have different functions than the cells that synthesize them [314]. There is also evidence for an immunosuppressive activity of these vesicles. G-MDSC-derived exosomes have been found to limit the proliferation of CD4^+^ T cells and the release of IFN-γ and to intensify the expansion of Tregs [315]. Glioma-derived exosomes have been found to increase the release of IL-10 and the production of arginase-1 by unstimulated CD14^+^ monocytic cells [316]. G-MDSC-derived exosomes are anti-inflammatory, reduce the number of Th1 and Th17 cells and suppress their differentiation [317].

### Microvesicles and oncosomes

Microvesicles (MVs) belong to a subpopulation of vesicles with a diameter of up to 1000 nm. Their release from cells, unlike exosomes, occurs directly from the cell membrane by budding into the extracellular space [301]. The term “oncosomes” refers to microvesicles that contain active oncogenes [318]. Due to the high heterogeneity of MVs, the term “large oncosomes” (LO) is also used to refer to MVs whose diameter exceeds 1000 nm (1-10 μm) [319]. MVs released by cancer cells may contain various proteins that regulate immune responses, including chemokine receptors, proinflammatory cytokines such as IL-1 or IL-6 and cytokine receptors such as TNFR1 [320]. Cancer cells, by releasing MVs, can disrupt epithelial cell morphology and promote EMT leading to tumor progression [321]. The cargo of LOs differs from other vesicles and includes factors associated with tumor progression [322]. Hypoxic tumor-derived microvesicles (TD-MVs) transfer TGF-β1 to NK cells, which inhibits the function of these cells by decreasing NKG2D receptor activity. An additional immunosuppressive effect on NK cells relates to the transport of miR-23a, which disrupts the expression of the CD107a antigen in these cells [322]. Tumor-derived microvesicles obtained from colorectal cancer cells promote the differentiation of blood monocytes into macrophages with mixed features of M1/M2 polarization, induce phosphorylation of STAT1 and STAT3 and change the secretion of cytokines such as IL-10, IL-12 and TNF [323]. MV release is also dependent on cytokines. Incubation of cell lines derived from various tumors with TNF-α has been found to enhance the release of MVs [324]. Leukemia cell-derived MVs have also been found to disrupt lymphocyte functions by inducing T cell exhaustion [325]. Adverse effects of TD-MVs on T cells have also been reported in cell lines isolated from some solid cancers. Tumor-derived MVs enhance the proliferation of Tregs, inhibit the activation and proliferation of CD8^+^ T cells and significantly increase TGF-β1 levels in the supernatants of Tregs [326].

**Fig. 4:**
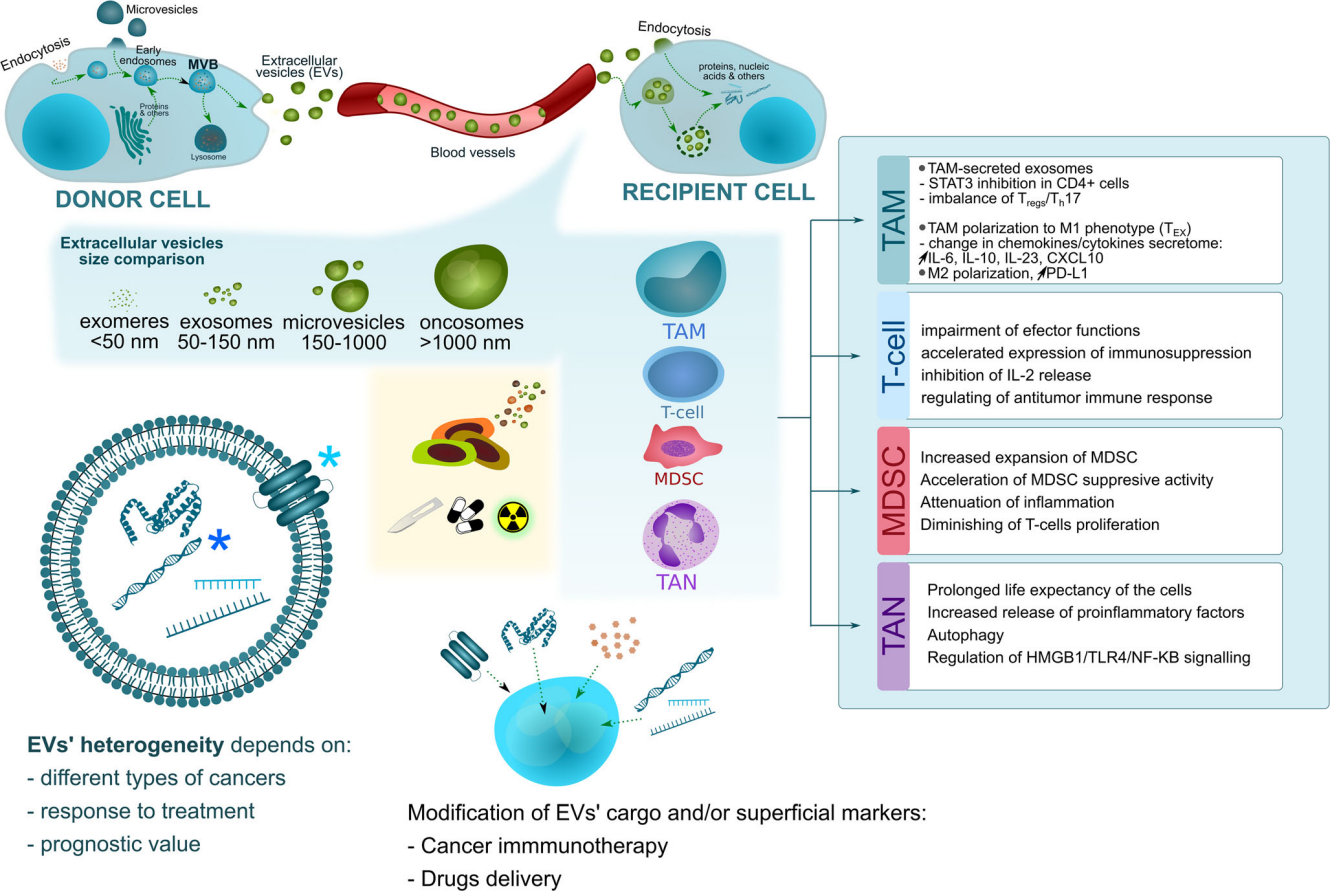
**Extracellular vesicles (EVs) actively secreted by cells transport various molecules, including proteins and genetic material, that are biologically active.** Communication between neoplastic and host cells using EVs may modify the metabolism of targeted cells and promote neoplastic development. Neoplastic vesicles may have immunosuppressive effects on the activity of immune cells. Specific cargo of these vesicles may promote cellular anergy and decrease the ability to present antigens by switching the immunophenotype of immune cells. Exosomes released into body fluids may facilitate biomarker discovery, with special emphasis on new markers for cancer progression and classification. As yet, little is known about molecular differences between chronic inflammation-associated cancers and sporadic cancers

One of the current hypotheses assumes modification of the activity of immunologically competent cells by exosomes [327]. Suppression of the immune response by cancer cells is thought to underlie tumor progression. Exosomes obtained from prostate cancer cells may limit the ability to present antigens and suppress the action of lymphocytes. These exosomes show the ability to inhibit the production of interleukin-2 by CD4^+^ T cells, to induce the expression of immunosuppressive CD73 antigen on dendritic cells and to suppress T cell activity in an adenosine-dependent manner affected by extracellular ATP/AMP [328]. The expression of several cytokine genes also seems to be dependent on exosomes, including exosome-induced overexpression of immunosuppressive factors, such as G-protein-coupled cannabinoid receptor 2 (CNR2, CB2), which has anti-inflammatory activity [329]. Pharmacological activation of this receptor in CRC by activation of AKT/PKB signaling and subsequent inhibition of GSK3β has been associated with tumor progression and an increase in tumor cell proliferation [330]. Hepatocellular carcinoma-derived exosomes can induce an immunosuppressive macrophage phenotype by upregulating PD-L1 ligand expression and overexpressing IL-10 [331]. With regard to prostate cancer, tumor-derived exosomes can lead to immune tolerance and activation of TGF-β signaling, which is related to progression of this tumor type [332]. Exosomes released by tumor cells show the ability to interact with normal cells of target tissues, and their effect on tumor biology has been found to depend on the specification of the maternal cells, the releasing vesicles and the cargo of the exosomes [333, 334]. Full-length proteins secreted in exosomes may be involved in the transmission of neoantigens during intercellular interactions, which can be crucial for the indirect presentation of antigens [335]. The role of exocytosis in the induction of tumor-induced immunosuppression is shown in Figure 4.

## Conclusions and perspectives

We discussed potential dependencies between well-known cancer risk factors and inflammation. Acute inflammation may promote changes that over time lead to cancer development. We have highlighted differences and similarities between acute phases of the immune response and chronic inflammation associated with tumor initiation. Particular attention has been paid to precancerous conditions that significantly increase the risk of cancer. We have discussed changes in immunophenotypes and secretory activities that occur in immune cells under conditions of chronic inflammation and cancer. Increased release of factors suppressing the immune response may be associated with a higher risk of progression toward chronic-inflammation-associated cancer.

**Fig. 5:**
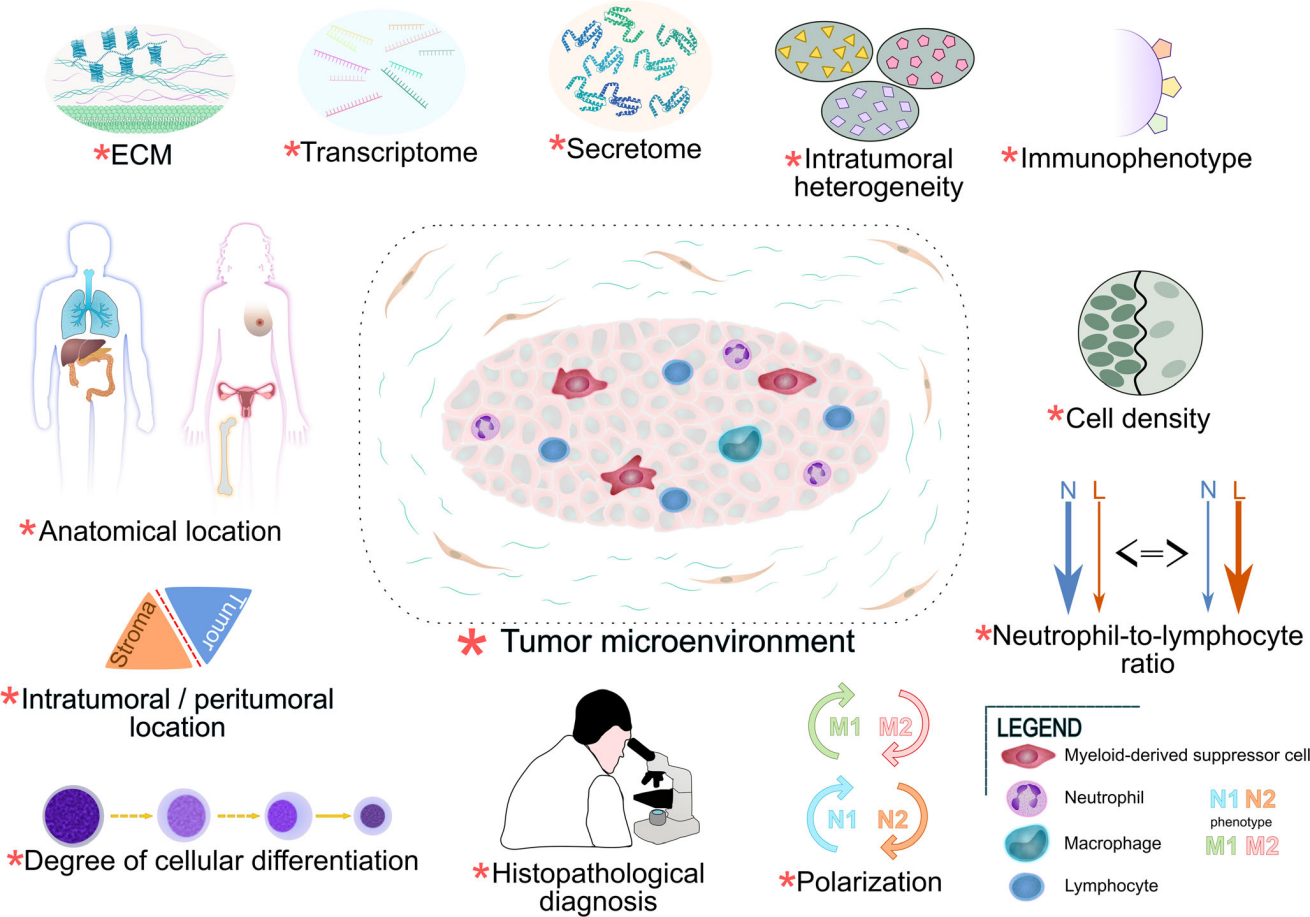
**The prognostic value of tumor-infiltrating lymphocytes (TILs) and other immune cells depends on various parameters at both cellular and tissue levels.** The tumor microenvironment (TME), the extracellular matrix (ECM) and the cancer cells affect the host’s adaptive immune response. On the other hand, the subtype, immunophenotype, microanatomical location and activity of inflammatory cells in the TME shape tumor immunity, which may have a pronounced effect on the patient’s clinical condition. The figure shows known molecular and tissue features (red stars) that have prognostic value due to their effect on inflammatory cells acting in the vicinity of the tumor.

An interesting issue is induced immunosuppression immediately after tumor resection (Fig. 3). In our model, we associate the subsequent deterioration of the immune response with the iatrogenic induced change in the heterogeneity of tumor cells and the secondary changes in their secretion activity. The immunophenotype of inflammatory cells and their metabolism may be modified by tumor cells through the release of exosomes: extracellular structures transporting biologically active factors regulating gene expression and metabolism of target cells (Fig. 4). It is a convenient way of modeling molecular changes during cancer development, both locally and systemically since exosomal cargos can be transported at considerable distances from the originating cell. Due to their stability, exosomes represent interesting sources of new biomarkers as well as tools for clinical application in selected diseases (Fig. 4). We also discussed changes in immune cells associated with prognosis (Fig. 5). Molecular changes in TILs are complex and depend on various factors.

Different mechanisms leading to suppression of the immune system may relate to diverse clinical outcomes. Primary immunodeficiencies are significantly different from tumors in both clinical characteristics and genotype conditions, which may explain the resulting discrepancies between these different groups of diseases. The limited functionality of the cellular response between chronic inflammation and cancer is possibly due to the participation of various immunosuppressive factors. In tumors, the participation of Treg cells is relatively pronounced. Their immunosuppressive activity may be associated with progression of the disease. The biological activity of some cytokines, which may have unspecified biological effects during carcinogenesis, seems to be particularly interesting. A more detailed assessment of these relationships and the importance of immunosuppression in the pathogenesis of tumors requires further research.
